# Effect of COVID-19 on infections associated with medical devices in critical care

**DOI:** 10.1186/s12879-023-08934-1

**Published:** 2024-01-22

**Authors:** Fredy Leonardo Carreño Hernández, Juanita Valencia Virguez, Juan Felipe González Vesga, María Lucía Castellanos, Gabriela Ruiz Beltrán, Laura Daniela Lorza Toquica, Carol Natalia Sánchez Gomez, Maria Valentina Stozitzky Ríos, Yenny Rocío Cárdenas Bolívar, Jorge Iván Alvarado Sanchez

**Affiliations:** 1 School of Medicine, Universidad de los Andes, Bogotá, D.C., Colombia; 2 Universidad de los Andes, Bogotá, Colombia; 3 Fundación Santa Fe de Bogotá, Bogotá, Colombia; 4 Fundación Santa Fe de Bogotá, Bogotá, Colombia; 5 Universidad Nacional de Colombia, Bogotá, Colombia

**Keywords:** COVID-19, Ventilation-associated pneumonia, Heatlhcare-associated infections, Sthaphylococcus auerus, Critically ill patients, Intensive care unit

## Abstract

**Objectives:**

This study explores the hypothesis that COVID-19 patients are at a heightened risk of healthcare-associated infections (HAIs) associated with medical device usage compared to non-COVID-19 patients. Our primary objective was to investigate the correlation between COVID-19 infection in ICU patients and subsequent HAIs following invasive medical device insertion. Additionally, we aim to assess the impact of SARS-CoV-2 infection on onset times concerning specific microorganisms and the type of medical device, providing valuable insights into this intricate relationship in intensive care settings.

**Methodology:**

A retrospective cohort study was conducted using ICU patient records at our hospital from 2020 to 2022. This investigation entailed evaluating the timing of HAIs while distinguishing between patients with and without SARS-CoV-2 infection. We identified and analyzed the type of isolation and infection attributed to the medical device while controlling for ICU duration and ventilator days using Cox regression.

**Results:**

Our study included 127 patients without SARS-CoV-2 infection and 140 patients with SARS-CoV-2 infection. The findings indicated a higher incidence of HAI caused by various microorganisms associated with any medical device in patients with SARS-CoV-2 (HR = 6.86; 95% CI-95%: 3.26–14.43; *p* < 0.01). After adjusting for ICU duration and ventilator days, a heightened frequency of HAIs persisted in SARS-CoV-2-infected individuals. However, a detailed examination of HAIs revealed that only ventilation-associated pneumonia (VAP) displayed a significant association (HR = 6.69; 95% CI: 2.59–17.31; *p* < 0.01). A statistically significant correlation between SARS-CoV-2 infection and the isolation of *S. aureus* was also observed (*p* = 0.034). The prevalence of *S. aureus* infection was notably higher in patients with SARS-CoV-2 (RR = 8.080; 95% CI: 1.052–62.068; *p* < 0.01).

**Conclusions:**

The frequency of pathogen isolates in invasive medical devices exhibited an association with SARS-CoV-2 infection. Critically ill patients with SARS-CoV-2 are more prone to developing early-onset VAP than those without SARS-CoV-2 infection.

**Supplementary Information:**

The online version contains supplementary material available at 10.1186/s12879-023-08934-1.

## Background

Healthcare-associated infections (HAIs) encompass infections that emerge within 48 h after hospitalization, within 30 days of receiving medical care, or up to 90 days following specific surgical procedures, regardless of the care setting [1]. HAIs are the sixth leading cause of mortality in high-income countries, with those linked to medical devices constituting a critical contributor to global morbidity and mortality, particularly within the intensive care unit (ICU), where mortality rates can soar up to 38.4% [2]. Prominent microorganisms responsible for device-associated HAIs include coagulase-negative *staphylococci*, *Staphylococcus aureus, Pseudomonas aeruginosa, Klebsiella pneumoniae, Enterococci, Candida* species, *Streptococci, and Escherichia coli*, among others [3], underscoring the substantial challenges posed by HAIs within patient care.

Within the realm of critical medicine, invasive medical devices play an indispensable role in patient management [4]. However, these devices entail an inherent risk of HAIs [5], exacerbating the crisis of antibiotic resistance, extending hospital stays, and amplifying mortality rates [4]. Furthermore, evidence suggests that ICU patients face heightened colonization pressure [6], rendering them 5 to 10 times more susceptible to HAIs than non-ICU patients [7]. In Colombia, data from the Epidemiological Bulletin by *Instututo Nacional de Salud* (INS) indicate that until week 08 of 2021, there were 1,357 cases of device-associated infections in ICUs, signifying a 40% surge in reported cases compared to the preceding year [8].

The global landscape was reshaped by the COVID-19 pandemic, impacting sectors such as the economy, health, environment, and education. The introduction of novel microorganisms, exemplified by SARS-CoV-2, disrupted existing paradigms, exacerbating severity, hospitalization, and intubation rates for patients [9]. Notably, patients afflicted with COVID-19 are predisposed to acquiring healthcare-associated bacterial infections, attributed to immunosuppression and increased invasive medical procedures [5]. The pandemic has further been linked to escalated rates of nosocomial infections involving multidrug-resistant microorganisms, spanning both ICU and non-ICU hospital settings [10], solidifying the intricate interplay between SARS-CoV-2 and HAIs.

However, the nexus between pathogen isolates in invasive medical devices and their interrelation with COVID-19 remains unclear. Moreover, the distinction between HAIs stemming from COVID-19 itself and those arising from the extensive invasive interventions necessitated by the disease remains ambiguous. Thus, this study seeks to discern potential disparities in isolates and HAIs among critically ill patients with COVID-19 and those without yet requiring medical devices, shedding light on a complex and poorly understood aspect of patient care.

## Methodology

### Overall study design

A retrospective observational analytical cohort study was carried out in which patients admitted to the Fundación Santa Fe de Bogotá ICU were followed up from admission to discharge or death within the period from December 1, 2020, to September 30, 2022. This research was approved by the ethics committee of the *Fundación Santa Fe de Bogotá* (FSFB) with code CEIS-14588-2022.

### Population

Utilizing data obtained from the ICU censuses at FSFB, we pinpointed patient records featuring the presence of at least one medical device—such as orotracheal intubation, bladder catheter, or central venous catheter. Within these records, patients were stratified based on their SARS-CoV-2 infection status as determined by PCR testing results. Subsequently, a randomized selection process was employed to assemble the cohort. Within this cohort, an exhaustive manual review of the corresponding medical records ensued. During this review, exclusion criteria were identified and applied. These criteria encompassed instances where the clinical record was either incomplete or inaccessible, the ICU stay duration was less than 24 h, medical devices were voluntarily withdrawn, patients were transferred either from another ICU or to another ICU, or the development of SARS-CoV-2 infection occurred during their ICU management.

#### Comparator

Critically ill patients without COVID-19 but require invasive medical devices.

#### Outcomes

##### Primary Outcome

We aimed to measure the time in days from ICU admission to the emergence of HAIs linked to medical devices. The HAIs were divided into three groups: ventilator-associated pneumonia (VAP), catheter-associated bloodstream infection (ITS-AC) and symptomatic catheter-associated urinary tract infection (ITSU-AC). We focused on establishing a potential connection between infection onset and the specific type of implicated microorganism. This assessment involved identifying both the underlying cause of the infection and the associated medical device.

##### Additional outcome

A comprehensive count of pathogen identifications was conducted, categorized by overall prevalence and specific device types. This analysis sought to reveal patterns and variations in HAI occurrence across different medical devices, providing a comprehensive grasp of the device utilization-infection development relationship.

### Clinical variables

For all enrolled subjects, key variables including age (measured in years), SARS-CoV-2 infection status (yes/no), duration of ICU stay (in days), utilization of specific medical devices such as central venous catheter, bladder catheter, and/or orotracheal intubation with or without tracheostomy, as well as the duration of usage for each mentioned medical device, and relevant medical history were extracted.

Regarding the outcomes, HAIs were defined by the presence of symptoms indicative of local or systemic infection, in conjunction with a confirmed positive culture for bacteremia associated with a central venous catheter, urinary tract infection linked to a bladder catheter, and ventilator-associated pneumonia (VAP) identified either by radiographic consolidation or by the presence of clinical symptoms if culture results were not available. In instances where a pathogen was identified through culture or film array but the patient remained asymptomatic, this was documented as pathogen isolation solely based on culture in the medical records and identification via film array. For isolates in each medical device, distinction was made for the resistance pattern with the bacteria *S. aureus* (methylcillin sensitive - MSSA/methylcillin resistant MRSA) and *K. pneumoniae* (with or without KPC), while for the general identification, only the type of pathogen was not distinguished by resistance.

The day of infection was meticulously recorded, stratified by both the type of medical device and the specific pathogen category, which encompassed gram-positive, gram-negative, fungal, and atypical microorganisms. Last, instances of patient mortality during their ICU stay, along with the corresponding dates of death, were meticulously documented.

### Statistical analysis

Quantitative data were summarized utilizing either means accompanied by their corresponding standard deviations or medians along with interquartile ranges, contingent upon the data distribution characteristics. Qualitative data were presented using frequencies and percentages, in accordance with the nature of the information. The quantitative data underwent assessment through T tests, while the analysis of qualitative data was performed employing chi-squared tests, each tailored to the specific data type, to derive meaningful insights from the dataset. For the bivariate analysis, the relative risk (RR) was computed to discern disparities in the frequency of identified pathogens between individuals diagnosed with and without SARS-CoV-2 infection.

A cumulative event analysis by Kaplan‒Meier survival curves between COVID-19 infection status was conducted in all cohorts to illustrate the occurrence of HAIs over time, considering all HAIs, individualized by medical device, and categorized by the type of pathogen. For those cumulative event analyses that showed statistically significant differences in all cohorts (*p* < 0.05), univariate and multivariate Cox regression analyses were employed to calculate the hazard ratio (HR), contrasting the risk rates for overall HAIs and specifically within outcomes exhibiting significant differences. The outcomes were used as independent variables. Additionally, the model was achieved while controlling for variables such as age, diabetes, smoking, lung disease, and duration of medical device utilization. These variables were chosen because they are highly associated with infections [11–13]. To choose the optimal Cox regression model across cohorts, the Akaike information criterion (AIC) with the least mean squared error was considered, and the assumptions were evaluated only for those models.

The assumption of a constant risk rate over time was evaluated through a global Schoenfeld test, and the models were examined for influential observations. Furthermore, to examine the potential effect of UCI days and ventilation days, a secondary inferential analysis was conducted involving randomized patient selection, which was adjusted for ICU stay duration and ventilation time. All statistical analyses were performed using RStudio. *p* < 0.05 was considered statistically significant.

## Results

### General characteristics of the patients

**Fig. 1:**
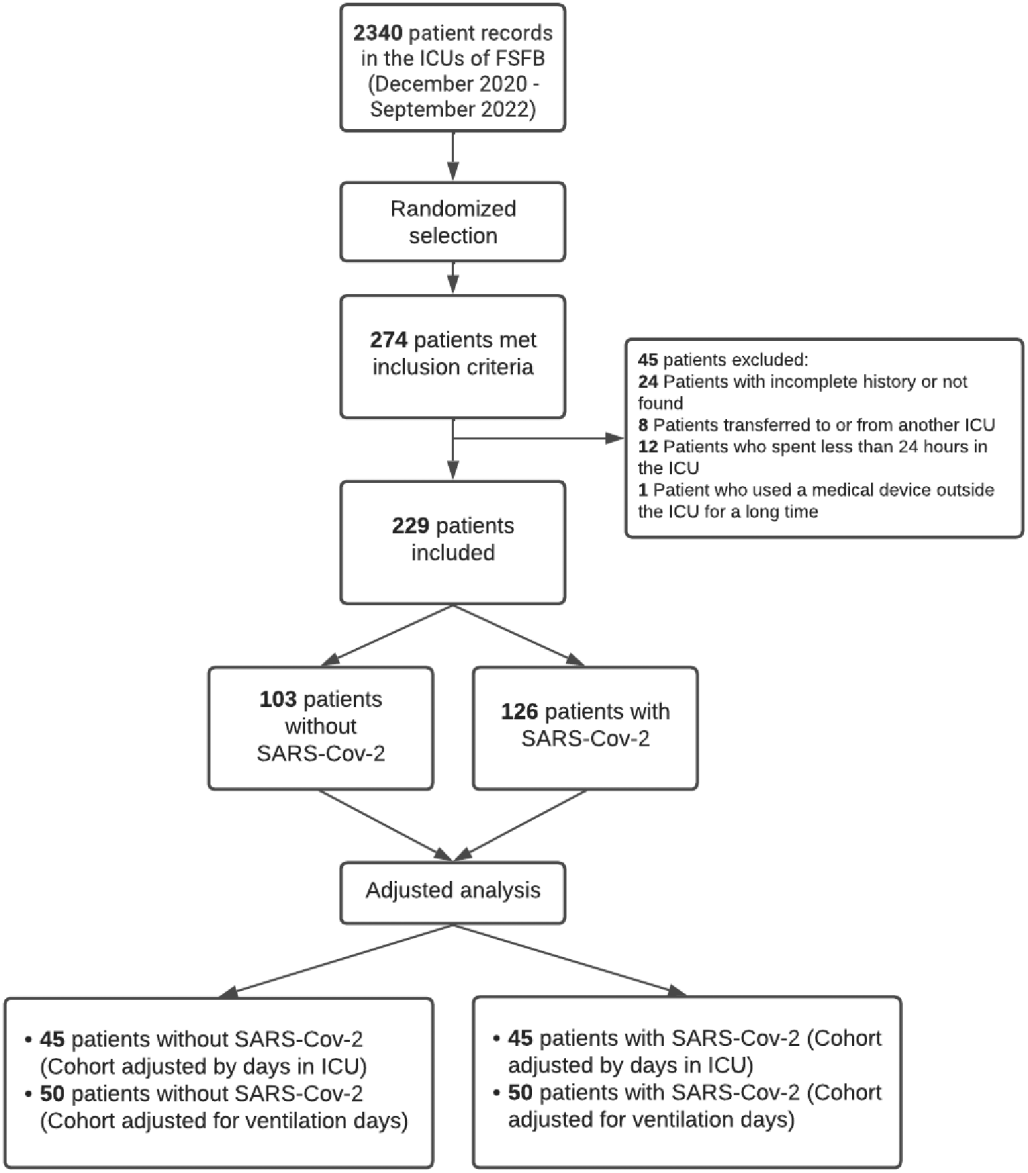
Flowchart for medical record selection

The research commenced using a dataset comprising 2,340 patients who were admitted to the FSFB-ICU between December 2020 and September 2022. Through a randomization process, 274 patients meeting the inclusion criteria were selected. Subsequently, exclusion criteria were applied, resulting in a final study population of 229 patients: 103 without SARS-CoV-2 infection and 126 with SARS-CoV-2 infection. The selection process is described in Fig. 1.

Analysis revealed that patients afflicted with COVID-19 had a median ICU stay of 12.5 days, whereas those without SARS-CoV-2 infection had a median stay of 5 days (*p* < 0.001). Bivariate and multivariate analyses were conducted, accompanied by graphical representation, for the cohort encompassing all patients and the two adjusted cohorts. Upon adjusting the analysis for ICU stay days, two distinct groups emerged: one comprised 45 patients for each category of SARS-CoV-2 infection. In the case of the cohort adjusted based on ventilation days, there were 50 patients in each group.

Across all three cohorts, statistical significance was observed only in terms of increased mortality among COVID-19 patients. Meanwhile, the significance of medical device usage duration was evident solely in the overall cohort and the cohort adjusted for ICU days. Detailed clinical data for all cohorts can be found in Table 1.

**Table 1:** Clinical characteristics of the patients

Clinical features	Total cohort			Cohort adjusted by days in ICU			Cohort adjusted for ventilation days		
	COVID-19	No COVID-19	*p*	COVID-19	No COVID-19	*p*	COVID-19	No COVID-19	*p*
Patients (n=)	129	103	-	45	45	-	50	50	-
Age (years) Median-IQR	68 [58–76]	64 [50.5–76]	0.065	69 [58–76]	68 [48–78]	0.37	69 [59– 77]	67 [51– 81]	0.95
Mortality (n= %)	120 (92%)	54 (52%)	< 0.01	44 (97%)	27 (60%)	< 0.0001	50 (100%)	36 (72%)	< 0.0001
Sex F (n= %)	41 (41%)	45 (43%)	0.11	18 (40%)	15 (33%)	0.66	16 (32%)	23 (46%)	0.21
Smoking (n= %)	14 (11%)	21 (20%)	0.07	5 (11%)	10 (22%)	0.25	5 (10%)	9 (18%)	0.38
Obesity (n= %)	21 (16%)	22 (21%)	0.46	10 (22%)	9 (20%)	1	10 (20%)	10 (22%)	1
Hypothyroidism (n= %)	29 (23%)	15 (15%)	0.14	7 (15%)	10 (22%)	0.59	10 (20%)	7 (14%)	0.54
Diabetes (n= %)	33 (26%)	15 (14%)	0.046	12 (26%)	7 (15%)	0.3	14 (28%)	7 (14%)	0.14
High blood pressure (n= %)	51 (4%)	50 (48%)	0.27	15 (33%)	22 (48%)	0.19	18 (32%)	25 (50%)	0.25
Chronic heart disease (n= %)	29 (23%)	31 (30%)	0.28	4 (8.9%)	12 (26%)	0.053	19 (38%)	11 (22%)	0.12
Lung disease (n= %)	21 (16%)	17 (16%)	1	6 (13%)	8 (17%)	0.77	9 (18%)	7 (14%)	0.78
Burn (n= %)	0	1 (1.6%)	-	0	1 (2.2%)	-	0	1 (2%)	-
Chronic Renal Disease (n= %)	10 (9.0%)	10 (9.7)	0.59	3 (6.7)	8 (17%)	0.19	3 (6%)	5 (10%)	0.71
Chronic liver disease (n= %)	12 (10%)	11 (10%)	1	4 (8.9%)	5 (11%)	1	6 (12%)	6 (12%)	1
Neurological disease (n= %)	12 (9.5%)	19 (18%)	0.076	1 (2.2%)	9 (20%)	0.018	4 (8%)	9 (18%)	0.23
Chronic Immunosuppression (n= %)	21 (16%)	9 (8.7%)	0.11	8 (17%)	4 (8.9%)	0.35	8 (16%)	2 (4%)	0.095
Neoplasm (n= %)	12 (9%)	15 (14%)	0.33	5 (11%)	8 (17%)	0.54	6 (12%)	8 (16%)	0.77
Use of central venous catheter (n= %)	123 (98%)	90 (87%)	< 0.0001	45 (100%)	43 (95%)	0.54	50 (100%)	46 (92%)	0.12
Central venous catheter days	13. 7 [6.73–20.8]	5 [2.72–11]	< 0.0001	12.4 [8.09–15]	8.97 [6.04–14.2]	0.09	8.04 [4.34–11.8]	5.84 [3.01–11.5]	0.63
Central venous catheter isolation (n= %)	33 (26%)	13 (12%)	0.012	9 (20%)	7 (15%)	0.78	5 (10%)	4 (10%)	1
ITS (n= %)	30 (23%)	8 (7.9%)	< 0.0001	9 (20%)	4 (8.9%)	0.23	5 (10%)	1 (2%)	0.2
Use of urinary catheter (n= %)	120 (95%)	86 (83%)	< 0.0001	43 (95%)	41 (91%)	0.67	50 (100%)	45 (90%)	0.06
Days with urinary catheter	14.6 [7.49–21.0]	5 [2.72–11]	< 0.0001	12.4 [8.10–15.4]	9 [6.08–13]	0.044	8.04 [4.50–11.8]	5.60 [3.16–11.5]	0.44
Urinary catheter isolation (n= %)	29 (23%)	6 (5.8%)	< 0.0001	8 (17%)	4 (8.9%)	0.35	6 (12%)	3 (6%)	0.48
ISTU AC (n= %)	19 (15%)	2 (1.9%)	0.0013	6 (13%)	1 (2.2%)	0.11	4 (8%)	1 (2%)	0.35
Use of intubation or tracheostomy (n= %)	118 (93%)	77 (74%)	< 0.0001	42 (93%)	37 (82%)	0.19	50 (100%)	50 (100%)	-
Días con intubación o traqueostomía	14.5 [7–21.0]	4 [2.33–9.20]	< 0.0001	11.0 [7.18–14.7]	8 [5.27–11.1]	0.014	6.91 [4.08–10.5]	5 [2.96–9.61]	0.12
Isolations in intubation or tracheostomy (n= %)	56 (44%)	10 (9.8%)	< 0.0001	21 (43%)	9 (20%)	0.013	14 (28%)	6 (12%)	0.08
VAP (n= %)	48 (36%)	8 (7.8%)	< 0.0001	18 (40%)	7 (15%)	0.018	13 (26%)	4 (8%)	0.03
gram-positive infection (n= %)	46 (36%)	8 (7.8%)	< 0.0001	11 (24%)	4 (8.9%)	0.089	7 (14%)	3 (6%)	0.31
gram-negative infection (n= %)	41 (32%)	10 (9.7%)	< 0.0001	10 (22%)	8 (17%)	0.79	8 (16%)	3 (6%)	0.2
Atypical Infection (n= %)	8 (6.3%)	2 (1.9%)	0.19	6 (13%)	2 (4.4%)	0.26	0	3 (6%)	0.24
Fungal Infection (n= %)	13 (10%)	2 (1.9%)	0.022	5 (11%)	1 (2.2%)	0.20	0	3 (6%)	0.24

IQR: Interquartile range

VAP: Ventilation associated pneumonia

### Identification and isolations

The predominant pathogen consistently identified in cases of SARS-CoV-2 infection was *K. pneumoniae*. Interestingly, *S. aureus* identification displayed an association with COVID-19 infection (*p* < 0.01), albeit with a wide confidence interval (RR = 8,08; 95%-CI: 1,05–62,06), but no similar association was observed through medical devices.

**Fig. 2:**
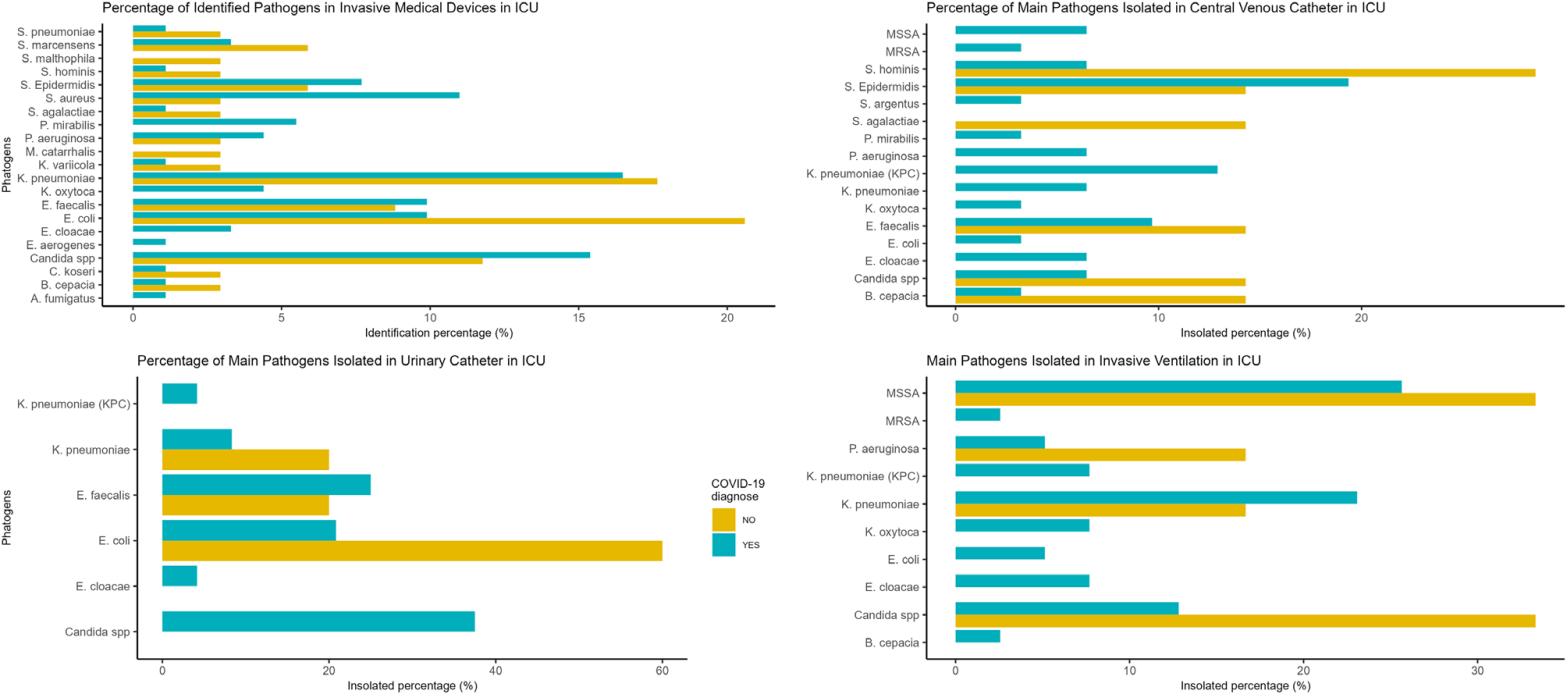
Microorganism identification and isolation percentageMSSA: Methicillin sensible Staphylococcus aureusMRSA: Methicillin resistant Staphylococcus aureus

Conversely, several pathogens, including *S. malthophila, P. mirabilis, M. catarrhalis, K. oxytoca, E. cloacae, E. aerogenes, and A. fumigatus*, yielded results that could not be reliably estimated. As such, establishing a definitive relationship between these pathogens and COVID-19 in any medical device was not feasible. For the remaining pathogens, no statistically significant correlation with COVID-19 infection was observed, and these findings are detailed in Table 2; Fig. 2.

**Table 2:** Association Measures between COVID 19 Infection and identifications of Microorganisms in Invasive Medical Devices in Critically ill Patients

Microorganism	RR	*P*	CI
*E.coli*	1.04	1.00	0.40–2.69
*S.aureus*	8.08	0.03 *	1.05–62.06
*K.pneumoniae*	2.02	0.18	0.81–5.02
*E.faecalis*	2.42	0.27	0.67–8.72
*Candida* spp.	2.63	0.12	0.88–7.81
*S.pneumoniae*	0.81	1.00	0.05–12.76
*S.marcensens*	1.21	1.00	0.21–7.12
*S.malthophila*	*Z*	0.92	*Z*
*S.hominis*	0.81	1.00	0.05–12.76
*S.epidermidis*	1.88	0.53	0.50–7.11
*S.agalactiae*	0.81	1.00	0.05–12.76
*P. miabilis*	*Z*	0.12	*Z*
*P.aeruginosa*	3.23	0.50	0.37–28.46
*M.catarrhalis*	*Z*	0.92	*Z*
*K.variicola*	0,81	1.00	0.05–12.76
*K.oxytoca*	*Z*	0.19	*Z*
*E.cloacae*	*Z*	0.33	*Z*
*E.aerogenes*	*Z*	1.00	*Z*
*C.koseri*	0,81	1.00	0.05–12.76
*B.cepacia*	0,81	1.00	0.05–12.76
*A.fumigatus*	*Z*	1.00	*Z*

*Z =* no estimable

RR = Relative Risk

* Statistically significant

The corresponding percentage of pathogen isolation varies by medical device. For the COVID-19 cohort, *S. aureus* methicillin sensible (MSSA) (25%), *K. pneumoniae* non-KPC (23%) and Candida spp. (13%) were the primary insolated pathogens for invasive ventilation, Candida spp. (37%), *E. faecalis* (25%) and *E. coli* (21%) were the primary insolated pathogens for urinary catheters, and *S. epidermidis* (19%), *K. pneumoniae* KPC (13%) and *E. faecalis* (25%) were the primary insolated pathogens for central venous catheters. Meanwhile, for non-COVID-19 cohort was MSSA (33%), Candida spp (33%) and *K. pneumoniae* non KPC (16%) where the primary insolated pathogens for invasive ventilation, while *E. coli* (66%), *E. faecalis* (20%) and *K. pneumoniae* non KPC (20%) for urinary catheter, *S. hominis* (28%) and similar percentages for the rest insolated pathogens (14%) in central venous catheter. Meanwhile, all isolation percentages are detailed in Fig. 2, and no statistical analysis was performed, as not enough data were available to test the association between pathogen isolation per medical device and COVID-19.

### HAIs

**Fig. 3:**
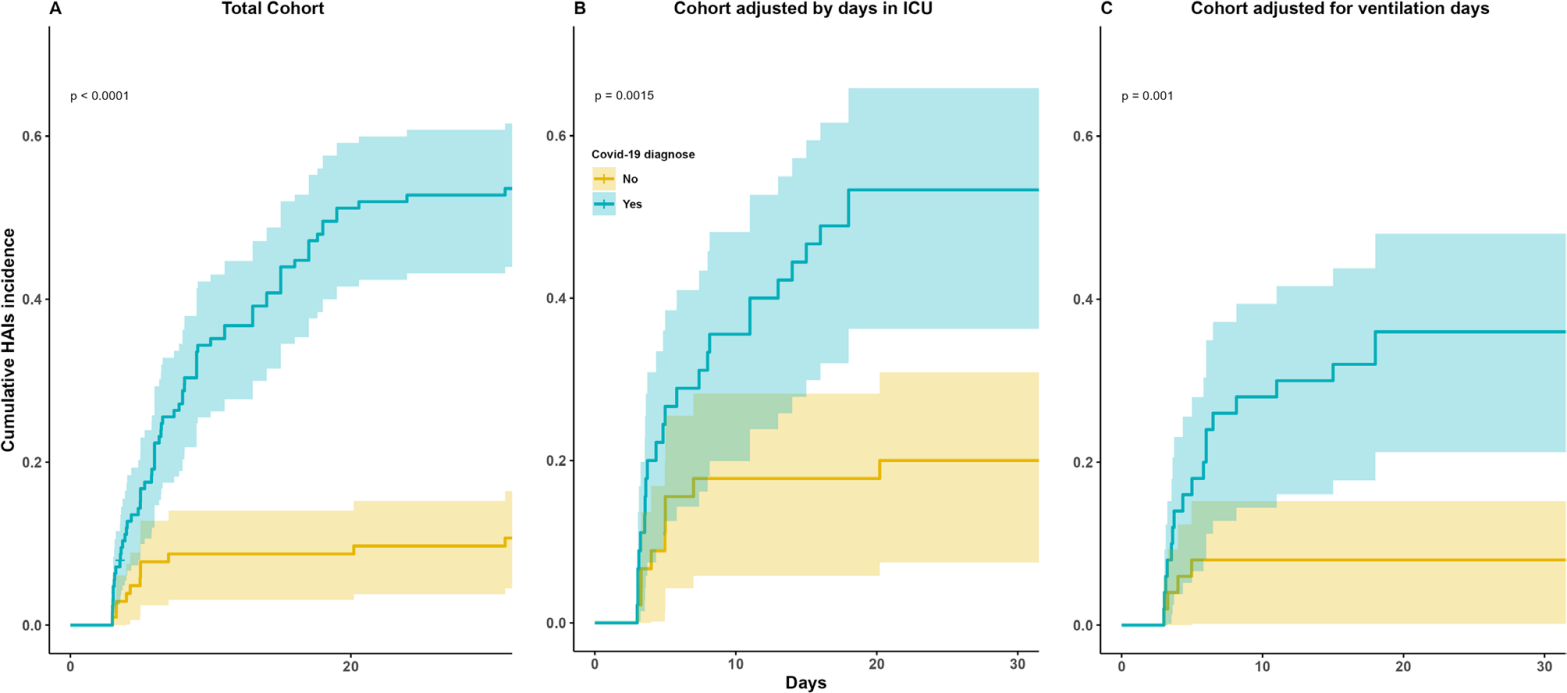
Cumulative incidence on any HAI´s based on COVID-19 by type of cohort

Regardless of the specific medical apparatus or type of pathogen, HAIs exhibited consistently higher occurrence rates among patients diagnosed with COVID-19 than among those without this diagnosis (*p* < 0.01), as illustrated in Fig. 3A, B and C. This trend persisted even when accounting for cohorts adjusted based on the duration of stay in the ICU and the duration of ventilation (*p* < 0.01).

In the univariable Cox regression analysis across the entire cohort, the hazard ratio (HR) for HAI among patients diagnosed with COVID-19 was 6.66 [95% CI: 3.52–12.62]. After adjusting for days spent in the ICU, the HR was 3.23 [95% CI: 1.50–6.97], and for the cohort adjusted for days on ventilation, the HR was 5.11 [95% CI: 1.73–15.11] (refer to Table 3). Notably, all three cohorts demonstrated statistically significant associations (*p* < 0.05) between days in the ICU and days on ventilation (Table 3).

**Table 3:** Univariable Cox regression model for HAI

Cohort	Variable	HR	95%-CI	*p*
Total Cohort	Covid-19	6.66	3.52–12.62	< 0.01 *
	Age	0.99	0.98–1.01	0.67
	Sex (male)	1.21	0.76–1.93	0.41
	days in ICU	1.29	1.02–1.04	< 0.01 *
	ventilation days	1.012	1.01–1.02	< 0.01 *
	Lung disease	0.62	0.31–1.23	0.16
	Diabetes	1.07	0.62–1.83	0.79
	Smoking	0.57	0.27–1.19	0.13
Cohort adjusted by days in ICU	Covid-19	3.235	1.50–6.97	< 0.01 *
	Age	0.99	0.97–1.01	0.46
	Sex (male)	0.97	0.48–1.97	0.93
	days in ICU	1.10	1.05–1.15	< 0.01 *
	ventilation days	1.12	1.06–1.19	< 0.01 *
	Lung disease	0.48	0.14–1.58	0.23
	Diabetes	1.37	0.63–2.96	0.41
	Smoking	0.27	0.06–1.16	0.07
Cohort adjusted for ventilation days	Covid-19	5.11	1.73–15.11	< 0.01 *
	Age	0.98	0.96–1.01	0.40
	Sex (male)	0.91	0.38–2.11	0.83
	days in ICU	1.03	1–1.06	0.04 *
	ventilation days	1.13	1.04–1.22	< 0.01 *
	Lung disease	0.22	0.03–1.67	0.15
	Diabetes	0.78	0.26–2.31	0.66
	Smoking	0.59	0.13–2.53	0.48

HR: Hazard Ratio

IC: confidence interval

ICU: intensive care unit

* Statical significant

In the context of multivariable Cox regression models encompassing all cohorts, even when accounting for factors such as sex, age, smoking, diabetes, and lung disease, the presence of COVID-19 remained statistically significant in relation to HAI (*p* < 0.05), consistent with the findings of the univariable models (see Table 4). The HR for the rest of the variables did not surpass 1.5. The cohort adjusted for days on ventilation displayed the optimal model for HAIs in general, boasting the lowest Akaike Information Criterion (AIC) and a minimal mean squared error of 183.16.

**Table 4:** Multivariable Cox regression model for HAI

Cohort	Variable	HR	95%-CI	*p*
Total Cohort	Covid-19	6.86	3.26–14.43	< 0.01 *
	Age	0.98	0.97–1.01	0.15
	Sex (male)	0.97	1.03–1.6	0.91
	days in ICU	1.02	1.00–1.04	< 0.01 *
	ventilation days	0.99	0.99–1.01	0.44
	Lung disease	0.68	0.33–1.39	0.29
	Diabetes	1.0247	0.57–1.83	0.93
	Smoking	1.1147	0.52–2.37	0.77
Cohort adjusted by days in ICU	Covid-19	5.02	1.82–13.81	< 0.01 *
	Age	0.96	0.94–0.99	0.04
	Sex (male)	0.86	0.38–1.93	0.71
	days in ICU	1.16	1.06–1.28	< 0.01 *
	ventilation days	1.01	0.93–1.10	0.71
	Lung disease	0.47	0.13–1.67	0.24
	Diabetes	0.83	0.33–2.09	0.70
	Smoking	1.12	0.24–5.12	0.88
Cohort adjusted for ventilation days	Covid-19	7.25	2.02–26.01	< 0.01 *
	Age	0.97	0.93–1.01	0.11
	Sex (male)	0.95	0.37–2.38	0.91
	days in ICU	1.03	0.99–1.08	0.09
	ventilation days	1.16	1.04–1.29	< 0.01 *
	Lung disease	0.22	0.028–1.81	0.16
	Diabetes	0.84	0.27–2.59	0.76
	Smoking	0.67	0.14–3.02	0.60

HR: Hazard Ratio

IC: confidence interval

ICU: intensive care unit

* Statical significant

Furthermore, the Schoenfeld global test for the optimal multivariable Cox regression model revealed no significant violations of the proportional hazards assumption (*p* = 0.2). It is noteworthy that the optimal Cox regression model identified 18 patients as having influential observations due to residual deviations greater than 1 or less than − 1.

### HAI by type of pathogen

**Fig. 4:**
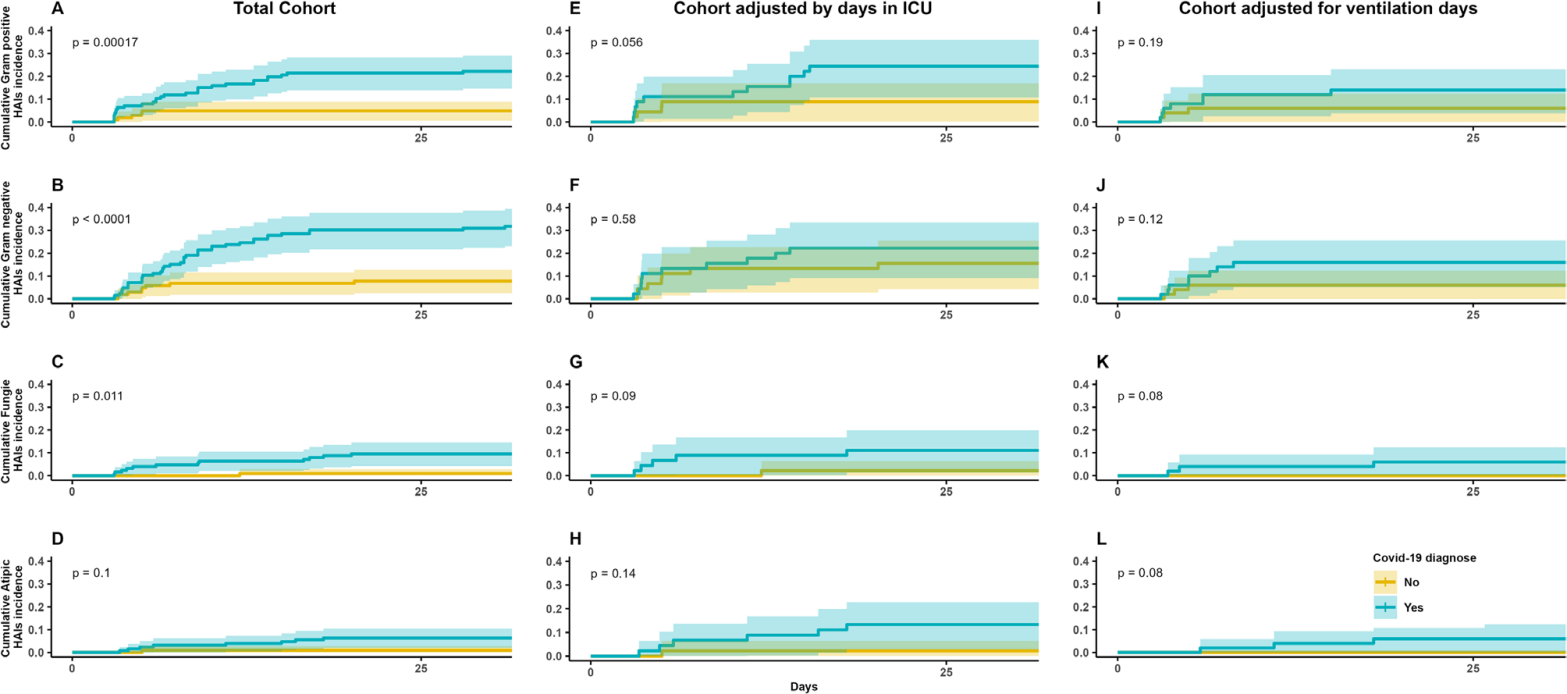
Cumulative incidence of HAI´s based on COVID-19 by type of causative pathogen and type of cohort

Kaplan‒Meier survival curves were plotted over time for the entire cohort, focusing on the emergence of HAIs attributed to gram-positive microorganisms (Fig. 4A), gram-negative microorganisms (Fig. 4B), and fungi (Fig. 4C). These analyses revealed a noteworthy divergence between patients with COVID-19 and those without, showing a notable increase in this category of infections among individuals diagnosed with COVID-19 (*p* < 0.01). However, such a discrepancy was not observed for infections caused by atypical microorganisms (Fig. 4D).

Upon examining the same Kaplan‒Meier survival curves for the two adjusted cohorts, no significant disparity was identified in the incidence of HAIs attributed to gram-positive, gram-negative, and fungal microorganisms between the two patient groups (*p* > 0.05), as demonstrated in Fig. 4E and L. As none of the outcomes exhibited substantial differences in the adjusted cohorts, there was no implementation of Cox regression analysis for HAIs categorized by the type of pathogen.

### HAI by medical device

**Fig. 5:**
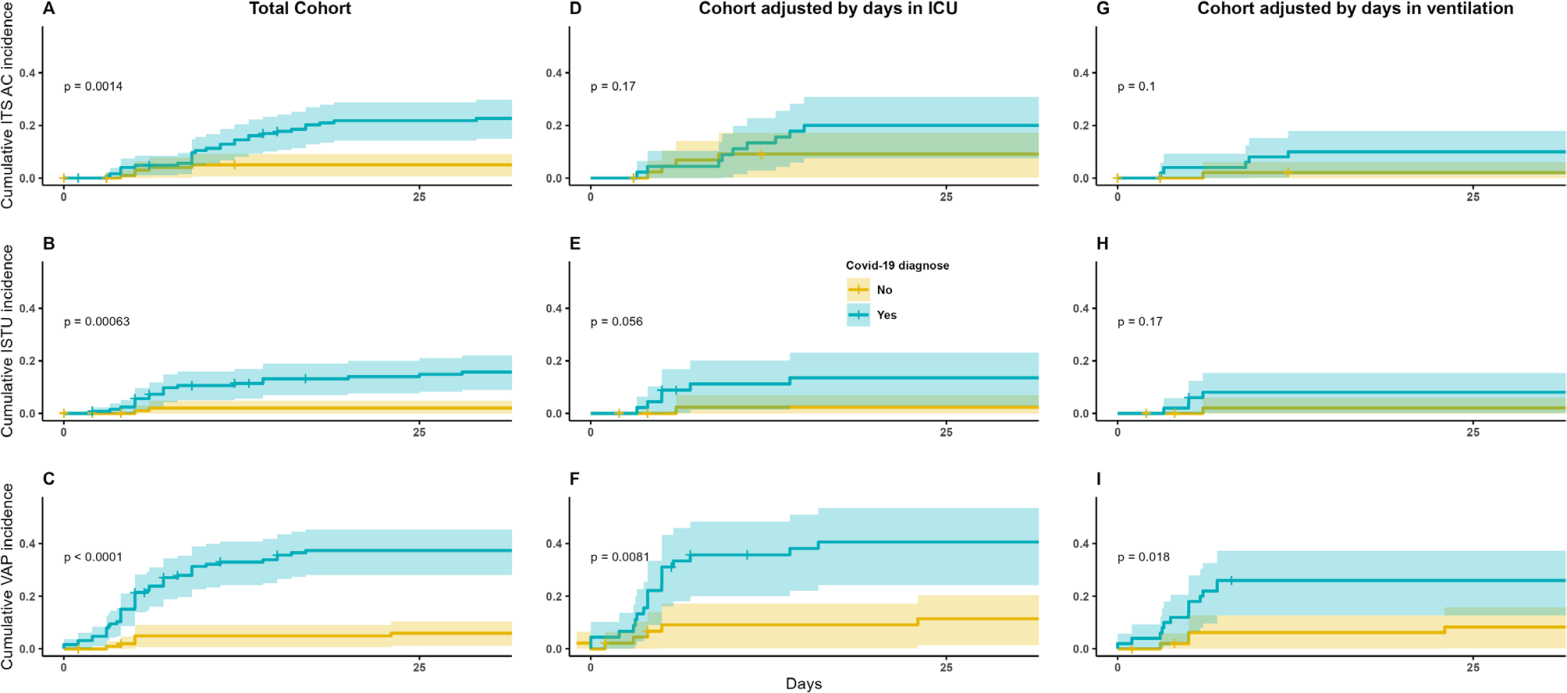
Cumulative incidence of HAI´s based on COVID-19 by medical device type and cohort type

When comparing the Kaplan‒Meier survival curves for healthcare-associated infection (HAI) events linked to different invasive devices within the entire cohort, significant differences in COVID-19 infection status were observed for ITS-AC, ISTU, and VAP, all with statistical significance (*p* < 0.01), as shown in Fig. 5A and B, and 5C, respectively. However, upon evaluating these same survival curves within both adjusted cohorts, statistically significant differences were not found for COVID-19 infection status in relation to ITS-AC and ISTU (*p* > 0.05), as depicted in Fig. 5D, E and G, and 5H. Nevertheless, the survival curves for VAP within the adjusted cohorts continued to manifest discrepancies in COVID-19 infection status (*p* < 0.02), as illustrated in Fig. 5F and I.

Only the univariable Cox regression analysis was conducted for VAP, yielding HR values of 5.78 [95%-CI: 2.72–12.25] for the total cohort, 3.074 [95%-CI: 1.28–7.33] for the cohort adjusted for days in the ICU, and 3.52 [95%-CI: 1.15–10.81] in the cohort adjusted for days on ventilation. All these HR values carried statistical significance (*p* < 0.05), as presented in Table 5, with analogous outcomes reflected in the multivariable Cox regression models within Table 6. While days spent in the ICU and on ventilation exhibited statistical significance within specific cohorts, none of these variables bore an HR greater than 1.5. Other variables included in the model did not demonstrate statistical significance.

**Table 5:** Univariable Cox regression model for VAP

Cohort	Variable	HR	95%-CI	*p*
Total Cohort	Covid-19	5.78	2.72–12.25	< 0.01 *
	Age	1	0.98–1.01	0.98
	Sex (male)	1.13	0.65–1.97	0.67
	days in ICU	1.03	1.02–1.04	< 0.01 *
	ventilation days	1.01	1.00–1.02	0.012 *
	Lung disease	0.47	0.19–1.19	0.11
	Diabetes	1.04	0.54–1.97	0.91
	Smoking	0.30	0.09–0.97	0.04 *
Cohort adjusted by days in ICU	Covid-19	3.074	1.28–7.33	0.02 *
	Age	0.99	0.97–1.02	0.82
	Sex (male)	1.05	0.46–2.34	0.90
	days in ICU	1.08	1.02–1.14	< 0.01 *
	ventilation days	1.09	1.02–1.17	0.01 *
	Lung disease	0.41	0.09–1.71	0.21
	Diabetes	1.11	0.44–2.77	0.82
	Smoking	0.18	0.02–1.33	0.09
Cohort adjusted for ventilation days	Covid-19	3.52	1.15–10.81	0.03 *
	Age	0.99	0.97–1.02	0.75
	Sex (male)	0.74	0.28–1.94	0.55
	days in ICU	1.03	0.99–1.06	0.06
	ventilation days	1.12	1.01–1.22	0.03 *
	Lung disease	0.29	0.04–2.18	0.22
	Diabetes	0.46	0.11–2.02	0.31
	Smoking	0.37	0.05–2.80	0.33

HR: Hazard Ratio

IC: confidence interval

ICU: intensive care unit

* Statical significant

**Table 6:** Multivariable Cox regression model for VAP

Cohort	Variable	HR	95%-CI	*p*
Total Cohort	Covid-19	6.69	2.59–17.31	< 0.01 *
	Age	0.99	0.97–1.01	0.74
	Sex (male)	0.89	0.49–1.61	0.70
	days in ICU	1.03	1.00–1.06	0.01
	ventilation days	0.98	0.95–1.01	0.22
	Lung disease	0.53	0.20–1.36	0.18
	Diabetes	0.91	0.45–1.83	0.80
	Smoking	0.59	0.18–1.93	0.36
Cohort adjusted by days in ICU	Covid-19	4.42	1.39–13.99	0.01*
	Age	0.98	0.95–1.01	0.45
	Sex (male)	1.15	0.47–2.81	0.83
	days in ICU	1.11	1.00–1.24	0.04 *
	ventilation days	1.00	0.90–1.11	0.93
	Lung disease	0.42	0.09–1.91	0.26
	Diabetes	0.76	0.26–2.23	0.62
	Smoking	0.53	0.06–4.20	0.55
Cohort adjusted for ventilation days	Covid-19	4.08	1.17–14.18	0.02 *
	Age	0.99	0.95–1.03	0.71
	Sex (male)	0.89	0.32–2.48	0.71
	days in ICU	1.03	0.99–1.08	0.12
	ventilation days	1.10	0.98–1.24	0.10
	Lung disease	0.27	0.03–2.20	0.22
	Diabetes	0.48	0.10–2.22	0.35
	Smoking	0.48	0.10–2.22	0.52

HR: Hazard Ratio

IC: confidence interval

ICU: intensive care unit

* Statical significant

Evaluating the assumptions, the best multivariable Cox regression model for VAP was determined based on the lowest Akaike Information Criterion (AIC), revealing a mean squared error of 152.36 for the cohort adjusted for days on ventilation. In this chosen optimal cohort, the global Schoenfeld test did not yield significant results (*p* = 0.44). The model was influenced by only 16 observations.

## Discussion

Our study revealed several key findings. Initially, there was a consistent pattern of increased mortality among COVID-19 patients across all examined cohorts. We also conducted an in-depth exploration of pathogen associations, identifying a noteworthy correlation between *S. aureus* and COVID-19 infection (*p* = 0.034). However, assessing several other pathogen associations proved challenging due to unreliable estimates. Additionally, the incidence of HAIs demonstrated a significant elevation in COVID-19 patients, irrespective of the type of pathogen involved (*p* < 0.01), even after adjusting for ICU and ventilation days. Analyzing the univariable Cox regression for HAIs related to VAP revealed a heightened HR within COVID-19 patients (3.52–5.78) across varying cohorts. Furthermore, the validity of COVID-19’s impact on VAP was confirmed through multivariable Cox regression models. These outcomes underscore the intricate nature of infection dynamics and emphasize the potential influence of COVID-19 on infection rates and associated outcomes.

The Centers for Disease Control and Prevention (CDC) in the United States conducted a study using the National Healthcare Safety Network. This study provided evidence of an increase in ventilator-associated events (VAEs) and methicillin-resistant *Staphylococcus aureus* (MRSA) bacteremia in 2021 compared to 2019 [14]. These findings bear some resemblance to the results presented in our own study. However, there are notable distinctions: we controlled for multiple confounding variables, such as the duration of ventilation, and due to our study design, we were able to assess potential causality. On the other hand, the same CDC study also reported increases in CLABSIs and CAUTIs. In contrast, our study did not identify these associations when controlling for confounding factors. It is worth noting that our results revealed the presence of certain pathogens that may be resistant to antibiotics, such as in the case of central venous catheters, which aligns with findings in the literature [14, 15]. Other studies focused on the pathogens identified, isolated, and responsible for infections have yielded results similar to ours. For example, one study found that the primary pathogens causing ventilator-associated pneumonia (VAP) in COVID-19 patients were *S. aureus* and *Enterobacteria* [16], which corresponds with our own study’s observation that Enterobacteria were the most frequently isolated pathogens in VAP cases.

These findings can be elucidated by considering the disease’s pathophysiology, particularly in relation to the generalized inflammatory response and the state of immunosuppression [17]. Invasive medical procedures are recognized as a risk factor for the development of both bacterial and fungal nosocomial infections [18]. The literature extensively describes how invasive medical devices breach mucosal or skin barriers, enabling pathogens to pass directly, thereby promoting nosocomial infections through their pathogenic mechanisms, including the formation of biofilms [19]. Furthermore, the increased severity of COVID-19 necessitates more complex medical support [20], which inherently elevates the risk of infection. It is crucial to emphasize that the duration of time spent on ventilation is a significant factor associated with infections in intensive care units [21], and given that COVID-19 patients typically require extended periods of ventilation, it is expected that this would contribute to the rise in HAIs.

It becomes clear that COVID-19 is likely causing a higher incidence of HAIs primarily because of prolonged hospital stays and increased use of invasive devices. This suggests that there might not be a distinct mechanism specific to COVID-19 for the generation of HAIs. However, VAP stands out as the most significant HAI that maintains a clear association with COVID-19, even after accounting for multiple confounding variables. This observation can be elucidated by considering that critically ill COVID-19 patients often develop ARDS. ARDS is known to heighten the risk of infections due to the exacerbated inflammatory response and cellular damage occurring in the alveoli [22, 23]. In simpler terms, VAP in critically ill COVID-19 patients can be attributed to ARDS rather than the virus itself.

It is crucial to recognize that viruses such as SARS-CoV-2 can hinder mucociliary clearance, a key mechanism in preventing respiratory infections, particularly those linked to *S. aureus* [24]. This correlation has been suggested to explain the heightened risk of healthcare-associated infections without medical device insertions or community-acquired infections [25, 26]. Consequently, it is plausible that ARDS caused by COVID-19 increases vulnerability to VAP by any pathogen. When coupled with impaired mucociliary clearance, there may be an even greater risk of VAP specifically by *S. aureus*. Our study has several strengths. First, the study’s selection and randomization process closely mimicked that of a clinical trial, enhancing its robustness. Second, patient characteristics in different study groups were well matched, minimizing potential biases, and any disparities in variables such as ICU stay duration or medical device usage were effectively addressed through cohort analysis.

Our study has some limitations. First, a limitation was the possibility of premature patient outcomes, such as death, occurring before identifying possible infections, which could result in the loss of infected or potentially infected patients. To mitigate this bias, Cox regression was employed during data analysis, reducing the impact of missing information and ensuring independence from the analyzed cohorts. However, it was noted that certain observations influenced the models, potentially leading to overestimation of HR. Second, another limitation stemmed from information bias due to the lack of evaluation of prior antibiotic therapies. This could lead to inaccuracies in variables. Third, it is noteworthy that all patients within COVID-19 cohorts develop ARDS since they all necessitate invasive ventilation, whereas the non-COVID-19 cohort experiences intubation for various reasons. To explore the hypothesis that VAP is induced by ARDS rather than directly by SARS-CoV-2, it is imperative to assemble a cohort of COVID-19 patients intubated for reasons other than ARDS. Finally, the study cautioned against generalizing its results to other institutions due to potential variations in ICU microbiota. More research is needed in this regard.

## Conclusion

In this study, we investigated the impact of SARS-CoV-2 infection on HAIs among patients in the FSFB-ICU. Our findings revealed that COVID-19 patients had significantly longer ICU stays, were associated with specific pathogens such as *K. pneumoniae* and *S. aureus* and exhibited a consistently higher prevalence of HAIs than non-COVID-19 patients. These trends persisted even after adjusting for ICU stay duration and ventilation days. Notably, VAP remained significantly linked to COVID-19. While this study sheds light on the elevated risk of HAIs in COVID-19 cases, further research is warranted to comprehensively understand these associations and develop tailored infection prevention strategies for critically ill patients, particularly those with COVID-19.

### Electronic supplementary material

Below is the link to the electronic supplementary material.

Supplementary Material 1

Supplementary Material 2

Supplementary Material 3

Supplementary Material 4

## Data Availability

The datasets used and/or analyzed during the current study are available from the corresponding author upon reasonable request.
