# Correction: Effect of COVID-19 on infections associated with medical devices in critical care

**DOI:** 10.1186/s12879-024-09070-0

**Published:** 2024-02-13

**Authors:** Fredy Leonardo Carreño Hernández, Juanita Valencia Virguez, Juan Felipe González Vesga, María Lucía Castellanos, Gabriela Ruiz Beltrán, Laura Daniela Lorza Toquica, Carol Natalia Sánchez Gomez, Maria Valentina Stozitzky Ríos, Yenny Rocío Cárdenas Bolívar, Jorge Iván Alvarado Sanchez

**Affiliations:** 1 Facultad de Medicina Universidad de los Andes, Bogotá, Colombia; 2 Universidad de los Andes, Bogotá, Colombia; 3 Fundación Santa Fe de Bogotá, Bogotá, Colombia; 4 Fundación Santa Fe de Bogotá, Bogotá, Colombia; 5 Universidad Nacional de Colombia, Bogotá, Colombia

**Correction: BMC Infectious Diseases 24, 110 (2024)**

**https://doi.org/10.1186/s12879-023-08934-1**

Following publication of the original article [1], we have been notified that Fig. 1 was published in Spanish instead of English.

The original article has been corrected.
