# Supplementary material for: Effect of COVID-19 on infections associated with medical devices in critical care
Source: BMC Infect Dis. 2024 Jan 22;24:110. doi: 10.1186/s12879-023-08934-1 (PMC10801999; doi:10.1186/s12879-023-08934-1)
Supplement: Supplementary file 1 — Supplementary Material 1 [file 12879_2023_8934_MOESM1_ESM.docx]

**Supplementary information**

1. Rstudio code: Análisis definitivo.doc
2. Complet data: IAAS Variables completas.xlsx
3. Cohort adjusted by days in ICU: cohorte ajustada por dias en UCI.xlsx
4. Cohort adjusted for ventilation days: Cohorte ajustada por dias de ventilación.xlsx

library(tidyr)

library(ggplot2)

library(readxl)

library(readr)

library(dplyr)

library(colorspace)

library(hrbrthemes)

library(datasets)

library(tibble)

library(ggpubr)

library(mgcv)

library(broom)

library(multcompView)

library(fs)

library(gmodels)

library(tidyverse)

library(caret)

library(nnet)

library(foreign)

library(reshape2)

library(ggpubr)

library(Hmisc)

library(lubridate)

library("survminer")

library("survival")

library ("epitools")

library(xfun)

#Extración de datos

setwd("C:/Users/lenovo/Documents/documentos importantes/Infecciónes UCI")

#Base de datos y análisis descriptivo

BASE <- read_excel("IAAS Variables completas.xlsx")

View(BASE)

describe(BASE)

BASE_2 <- read_excel("cohorte ajustada por dias en UCI.xlsx")

View(BASE_2)

describe(BASE_2)

BASE_3 <- read_excel("cohorte ajustada por dias de ventilación.xlsx")

View(BASE_3)

describe(BASE_3)

#Exclusión

BASE <- subset(BASE, INCLUIDO == "SI")

#Redefinición de terminos

BASE['DESENLACE'][BASE['DESENLACE'] == 'MUERTO'] <- '1'

BASE['DESENLACE'][BASE['DESENLACE'] == 'VIVO'] <- '0'

BASE$DESENLACE <- as.numeric(as.character(BASE$DESENLACE))

BASE$DÍAS_UCI <- as.numeric(as.character(BASE$DÍAS_UCI))

#Descripción general cohorte no ajustada

CrossTable(BASE$COVID, BASE$DESENLACE)

chisq.test(BASE$COVID, BASE$DESENLACE)

summary_1 <- BASE %>%

group_by(COVID) %>%

dplyr::summarize(mean = mean(EDAD, na.rm = TRUE),

sd = sd(EDAD, na.rm = TRUE),

median = median(EDAD, na.rm = TRUE),

Q1 = quantile(EDAD, probs = c(0.25), na.rm = TRUE),

Q3 = quantile(EDAD, probs = c(0.75), na.rm = TRUE))

summary_1

wilcox.test(EDAD ~ COVID, data=BASE)

CrossTable(BASE$COVID, BASE$SEXO)

chisq.test(BASE$COVID, BASE$SEXO)

CrossTable(BASE$COVID, BASE$Tabaquismo)

chisq.test(BASE$COVID, BASE$Tabaquismo)

CrossTable(BASE$COVID, BASE$Obesidad)

chisq.test(BASE$COVID, BASE$Obesidad)

CrossTable(BASE$COVID, BASE$Hipotiroidismo)

chisq.test(BASE$COVID, BASE$Hipotiroidismo)

CrossTable(BASE$COVID, BASE$Diabetes)

chisq.test(BASE$COVID, BASE$Diabetes)

CrossTable(BASE$COVID, BASE$HipertensionArterial)

chisq.test(BASE$COVID, BASE$HipertensionArterial)

CrossTable(BASE$COVID, BASE$ECardiacaCronica)

chisq.test(BASE$COVID, BASE$ECardiacaCronica)

CrossTable(BASE$COVID, BASE$Neumopatía)

chisq.test(BASE$COVID, BASE$Neumopatía)

CrossTable(BASE$COVID, BASE$Quemaduras)

chisq.test(BASE$COVID, BASE$Quemaduras)

CrossTable(BASE$COVID, BASE$ERenalCronica)

chisq.test(BASE$COVID, BASE$ERenalCronica)

CrossTable(BASE$COVID, BASE$EHepatica)

chisq.test(BASE$COVID, BASE$EHepatica)

CrossTable(BASE$COVID, BASE$ENeurologica)

chisq.test(BASE$COVID, BASE$ENeurologica)

CrossTable(BASE$COVID, BASE$Inmunosupresióncrónica)

chisq.test(BASE$COVID, BASE$Inmunosupresióncrónica)

CrossTable(BASE$COVID, BASE$NeoplasiaMalignaACTIVA)

chisq.test(BASE$COVID, BASE$NeoplasiaMalignaACTIVA)

CrossTable(BASE$COVID, BASE$CATETER_VASCULAR)

chisq.test(BASE$COVID, BASE$CATETER_VASCULAR)

BASE$días_cateter <- time_length(BASE$FECHA_FINAL_CATETER - BASE$FECHA_CATETER, unit="days")

summary_2 <- BASE %>%

group_by(COVID) %>%

dplyr::summarize(mean = mean(días_cateter, na.rm = TRUE),

sd = sd(días_cateter, na.rm = TRUE),

median = median(días_cateter, na.rm = TRUE),

Q1 = quantile(días_cateter, probs = c(0.25), na.rm = TRUE),

Q3 = quantile(días_cateter, probs = c(0.75), na.rm = TRUE))

summary_2

wilcox.test(días_cateter ~ COVID, data=BASE)

CrossTable(BASE$COVID, BASE$HEMOCULTIVO_POSITIVO)

chisq.test(BASE$COVID, BASE$HEMOCULTIVO_POSITIVO)

CrossTable(BASE$COVID, BASE$ITS_AC)

chisq.test(BASE$COVID, BASE$ITS_AC)

CrossTable(BASE$COVID, BASE$USO_SONDA)

chisq.test(BASE$COVID, BASE$USO_SONDA)

BASE$días_SONDA <- time_length(BASE$FECHA_FINAL_SONDA - BASE$FECHA_SONDA, unit="days")

summary_3 <- BASE %>%

group_by(COVID) %>%

dplyr::summarize(mean = mean(días_SONDA, na.rm = TRUE),

sd = sd(días_SONDA, na.rm = TRUE),

median = median(días_SONDA, na.rm = TRUE),

Q1 = quantile(días_SONDA, probs = c(0.25), na.rm = TRUE),

Q3 = quantile(días_SONDA, probs = c(0.75), na.rm = TRUE))

summary_3

wilcox.test(días_SONDA ~ COVID, data=BASE)

CrossTable(BASE$COVID, BASE$UROCULTIVO_POSITIVO)

chisq.test(BASE$COVID, BASE$UROCULTIVO_POSITIVO)

CrossTable(BASE$COVID, BASE$ISTU_AC)

chisq.test(BASE$COVID, BASE$ISTU_AC)

CrossTable(BASE$COVID, BASE$USO_OROTRAQUEAL_TRAQUEOSTOMIA)

chisq.test(BASE$COVID, BASE$USO_OROTRAQUEAL_TRAQUEOSTOMIA)

BASE$días_INTUBACIÓN <- time_length(BASE$FECHA_FINAL_OROTRAQUEAL_TRAQUEOSTOMIA - BASE$FECHA_OROTRAQUEAL_TRAQUEOSTOMIA, unit="days")

summary_4 <- BASE %>%

group_by(COVID) %>%

dplyr::summarize(mean = mean(días_INTUBACIÓN, na.rm = TRUE),

sd = sd(días_INTUBACIÓN, na.rm = TRUE),

median = median(días_INTUBACIÓN, na.rm = TRUE),

Q1 = quantile(días_INTUBACIÓN, probs = c(0.25), na.rm = TRUE),

Q3 = quantile(días_INTUBACIÓN, probs = c(0.75), na.rm = TRUE))

summary_4

wilcox.test(días_INTUBACIÓN ~ COVID, data=BASE)

CrossTable(BASE$COVID, BASE$CULTIVO_NEUMONIA_POSITIVAS)

chisq.test(BASE$COVID, BASE$CULTIVO_NEUMONIA_POSITIVAS)

CrossTable(BASE$COVID, BASE$NEU)

chisq.test(BASE$COVID, BASE$NEU)

CrossTable(BASE$COVID, BASE$IAAS_GRAM_POS)

chisq.test(BASE$COVID, BASE$IAAS_GRAM_POS)

CrossTable(BASE$COVID, BASE$IAAS_GRAM_NEG)

chisq.test(BASE$COVID, BASE$IAAS_GRAM_NEG)

CrossTable(BASE$COVID, BASE$IAAS_ATIPICOS)

chisq.test(BASE$COVID, BASE$IAAS_ATIPICOS)

CrossTable(BASE$COVID, BASE$IAAS_HONGOS)

chisq.test(BASE$COVID, BASE$IAAS_HONGOS)

CrossTable(BASE_2$COVID, BASE_2$DESENLACE)

chisq.test(BASE_2$COVID, BASE_2$DESENLACE)

summary_1 <- BASE_2 %>%

group_by(COVID) %>%

dplyr::summarize(mean = mean(EDAD, na.rm = TRUE),

sd = sd(EDAD, na.rm = TRUE),

median = median(EDAD, na.rm = TRUE),

Q1 = quantile(EDAD, probs = c(0.25), na.rm = TRUE),

Q3 = quantile(EDAD, probs = c(0.75), na.rm = TRUE))

summary_1

wilcox.test(EDAD ~ COVID, data=BASE_2)

CrossTable(BASE_2$COVID, BASE_2$SEXO)

chisq.test(BASE_2$COVID, BASE_2$SEXO)

CrossTable(BASE_2$COVID, BASE_2$Tabaquismo)

chisq.test(BASE_2$COVID, BASE_2$Tabaquismo)

CrossTable(BASE_2$COVID, BASE_2$Obesidad)

chisq.test(BASE_2$COVID, BASE_2$Obesidad)

CrossTable(BASE_2$COVID, BASE_2$Hipotiroidismo)

chisq.test(BASE_2$COVID, BASE_2$Hipotiroidismo)

CrossTable(BASE_2$COVID, BASE_2$Diabetes)

chisq.test(BASE_2$COVID, BASE_2$Diabetes)

CrossTable(BASE_2$COVID, BASE_2$HipertensionArterial)

chisq.test(BASE_2$COVID, BASE_2$HipertensionArterial)

CrossTable(BASE_2$COVID, BASE_2$ECardiacaCronica)

chisq.test(BASE_2$COVID, BASE_2$ECardiacaCronica)

CrossTable(BASE_2$COVID, BASE_2$Neumopatía)

chisq.test(BASE_2$COVID, BASE_2$Neumopatía)

CrossTable(BASE_2$COVID, BASE_2$Quemaduras)

chisq.test(BASE_2$COVID, BASE_2$Quemaduras)

CrossTable(BASE_2$COVID, BASE_2$ERenalCronica)

chisq.test(BASE_2$COVID, BASE_2$ERenalCronica)

CrossTable(BASE_2$COVID, BASE_2$EHepatica)

chisq.test(BASE_2$COVID, BASE_2$EHepatica)

CrossTable(BASE_2$COVID, BASE_2$ENeurologica)

chisq.test(BASE_2$COVID, BASE_2$ENeurologica)

CrossTable(BASE_2$COVID, BASE_2$Inmunosupresióncrónica)

chisq.test(BASE_2$COVID, BASE_2$Inmunosupresióncrónica)

CrossTable(BASE_2$COVID, BASE_2$NeoplasiaMalignaACTIVA)

chisq.test(BASE_2$COVID, BASE_2$NeoplasiaMalignaACTIVA)

CrossTable(BASE_2$COVID, BASE_2$CATETER_VASCULAR)

chisq.test(BASE_2$COVID, BASE_2$CATETER_VASCULAR)

BASE_2$días_cateter <- time_length(BASE_2$FECHA_FINAL_CATETER - BASE_2$FECHA_CATETER, unit="days")

summary_2 <- BASE_2 %>%

group_by(COVID) %>%

dplyr::summarize(mean = mean(días_cateter, na.rm = TRUE),

sd = sd(días_cateter, na.rm = TRUE),

median = median(días_cateter, na.rm = TRUE),

Q1 = quantile(días_cateter, probs = c(0.25), na.rm = TRUE),

Q3 = quantile(días_cateter, probs = c(0.75), na.rm = TRUE))

summary_2

wilcox.test(días_cateter ~ COVID, data=BASE_2)

CrossTable(BASE_2$COVID, BASE_2$HEMOCULTIVO_POSITIVO)

chisq.test(BASE_2$COVID, BASE_2$HEMOCULTIVO_POSITIVO)

CrossTable(BASE_2$COVID, BASE_2$ITS_AC)

chisq.test(BASE_2$COVID, BASE_2$ITS_AC)

CrossTable(BASE_2$COVID, BASE_2$USO_SONDA)

chisq.test(BASE_2$COVID, BASE_2$USO_SONDA)

BASE_2$días_SONDA <- time_length(BASE_2$FECHA_FINAL_SONDA - BASE_2$FECHA_SONDA, unit="days")

summary_3 <- BASE_2 %>%

group_by(COVID) %>%

dplyr::summarize(mean = mean(días_SONDA, na.rm = TRUE),

sd = sd(días_SONDA, na.rm = TRUE),

median = median(días_SONDA, na.rm = TRUE),

Q1 = quantile(días_SONDA, probs = c(0.25), na.rm = TRUE),

Q3 = quantile(días_SONDA, probs = c(0.75), na.rm = TRUE))

summary_3

wilcox.test(días_SONDA ~ COVID, data=BASE_2)

CrossTable(BASE_2$COVID, BASE_2$UROCULTIVO_POSITIVO)

chisq.test(BASE_2$COVID, BASE_2$UROCULTIVO_POSITIVO)

CrossTable(BASE_2$COVID, BASE_2$ISTU_AC)

chisq.test(BASE_2$COVID, BASE_2$ISTU_AC)

CrossTable(BASE_2$COVID, BASE_2$USO_OROTRAQUEAL_TRAQUEOSTOMIA)

chisq.test(BASE_2$COVID, BASE_2$USO_OROTRAQUEAL_TRAQUEOSTOMIA)

BASE_2$días_INTUBACIÓN <- time_length(BASE_2$FECHA_FINAL_OROTRAQUEAL_TRAQUEOSTOMIA - BASE_2$FECHA_OROTRAQUEAL_TRAQUEOSTOMIA, unit="days")

summary_4 <- BASE_2 %>%

group_by(COVID) %>%

dplyr::summarize(mean = mean(días_INTUBACIÓN, na.rm = TRUE),

sd = sd(días_INTUBACIÓN, na.rm = TRUE),

median = median(días_INTUBACIÓN, na.rm = TRUE),

Q1 = quantile(días_INTUBACIÓN, probs = c(0.25), na.rm = TRUE),

Q3 = quantile(días_INTUBACIÓN, probs = c(0.75), na.rm = TRUE))

summary_4

wilcox.test(días_INTUBACIÓN ~ COVID, data=BASE_2)

CrossTable(BASE_2$COVID, BASE_2$CULTIVO_NEUMONIA_POSITIVAS)

chisq.test(BASE_2$COVID, BASE_2$CULTIVO_NEUMONIA_POSITIVAS)

CrossTable(BASE_2$COVID, BASE_2$NEU)

chisq.test(BASE_2$COVID, BASE_2$NEU)

CrossTable(BASE_2$COVID, BASE_2$IAAS_GRAM_POS)

chisq.test(BASE_2$COVID, BASE_2$IAAS_GRAM_POS)

CrossTable(BASE_2$COVID, BASE_2$IAAS_GRAM_NEG)

chisq.test(BASE_2$COVID, BASE_2$IAAS_GRAM_NEG)

CrossTable(BASE_2$COVID, BASE_2$IAAS_ATIPICOS)

chisq.test(BASE_2$COVID, BASE_2$IAAS_ATIPICOS)

CrossTable(BASE_2$COVID, BASE_2$IAAS_HONGOS)

chisq.test(BASE_2$COVID, BASE_2$IAAS_HONGOS)

CrossTable(BASE_3$COVID, BASE_3$DESENLACE)

chisq.test(BASE_3$COVID, BASE_3$DESENLACE)

summary_1 <- BASE_3 %>%

group_by(COVID) %>%

dplyr::summarize(mean = mean(EDAD, na.rm = TRUE),

sd = sd(EDAD, na.rm = TRUE),

median = median(EDAD, na.rm = TRUE),

Q1 = quantile(EDAD, probs = c(0.25), na.rm = TRUE),

Q3 = quantile(EDAD, probs = c(0.75), na.rm = TRUE))

summary_1

wilcox.test(EDAD ~ COVID, data=BASE_3)

CrossTable(BASE_3$COVID, BASE_3$SEXO)

chisq.test(BASE_3$COVID, BASE_3$SEXO)

CrossTable(BASE_3$COVID, BASE_3$Tabaquismo)

chisq.test(BASE_3$COVID, BASE_3$Tabaquismo)

CrossTable(BASE_3$COVID, BASE_3$Obesidad)

chisq.test(BASE_3$COVID, BASE_3$Obesidad)

CrossTable(BASE_3$COVID, BASE_3$Hipotiroidismo)

chisq.test(BASE_3$COVID, BASE_3$Hipotiroidismo)

CrossTable(BASE_3$COVID, BASE_3$Diabetes)

chisq.test(BASE_3$COVID, BASE_3$Diabetes)

CrossTable(BASE_3$COVID, BASE_3$HipertensionArterial)

chisq.test(BASE_3$COVID, BASE_3$HipertensionArterial)

CrossTable(BASE_3$COVID, BASE_3$ECardiacaCronica)

chisq.test(BASE_3$COVID, BASE_3$ECardiacaCronica)

CrossTable(BASE_3$COVID, BASE_3$Neumopatía)

chisq.test(BASE_3$COVID, BASE_3$Neumopatía)

CrossTable(BASE_3$COVID, BASE_3$Quemaduras)

chisq.test(BASE_3$COVID, BASE_3$Quemaduras)

CrossTable(BASE_3$COVID, BASE_3$ERenalCronica)

chisq.test(BASE_3$COVID, BASE_3$ERenalCronica)

CrossTable(BASE_3$COVID, BASE_3$EHepatica)

chisq.test(BASE_3$COVID, BASE_3$EHepatica)

CrossTable(BASE_3$COVID, BASE_3$ENeurologica)

chisq.test(BASE_3$COVID, BASE_3$ENeurologica)

CrossTable(BASE_3$COVID, BASE_3$Inmunosupresióncrónica)

chisq.test(BASE_3$COVID, BASE_3$Inmunosupresióncrónica)

CrossTable(BASE_3$COVID, BASE_3$NeoplasiaMalignaACTIVA)

chisq.test(BASE_3$COVID, BASE_3$NeoplasiaMalignaACTIVA)

CrossTable(BASE_3$COVID, BASE_3$CATETER_VASCULAR)

chisq.test(BASE_3$COVID, BASE_3$CATETER_VASCULAR)

BASE_3$días_cateter <- time_length(BASE_3$FECHA_FINAL_CATETER - BASE_3$FECHA_CATETER, unit="days")

summary_2 <- BASE_3 %>%

group_by(COVID) %>%

dplyr::summarize(mean = mean(días_cateter, na.rm = TRUE),

sd = sd(días_cateter, na.rm = TRUE),

median = median(días_cateter, na.rm = TRUE),

Q1 = quantile(días_cateter, probs = c(0.25), na.rm = TRUE),

Q3 = quantile(días_cateter, probs = c(0.75), na.rm = TRUE))

summary_2

wilcox.test(días_cateter ~ COVID, data=BASE_3)

CrossTable(BASE_3$COVID, BASE_3$HEMOCULTIVO_POSITIVO)

chisq.test(BASE_3$COVID, BASE_3$HEMOCULTIVO_POSITIVO)

CrossTable(BASE_3$COVID, BASE_3$ITS_AC)

chisq.test(BASE_3$COVID, BASE_3$ITS_AC)

CrossTable(BASE_3$COVID, BASE_3$USO_SONDA)

chisq.test(BASE_3$COVID, BASE_3$USO_SONDA)

BASE_3$días_SONDA <- time_length(BASE_3$FECHA_FINAL_SONDA - BASE_3$FECHA_SONDA, unit="days")

summary_3 <- BASE_3 %>%

group_by(COVID) %>%

dplyr::summarize(mean = mean(días_SONDA, na.rm = TRUE),

sd = sd(días_SONDA, na.rm = TRUE),

median = median(días_SONDA, na.rm = TRUE),

Q1 = quantile(días_SONDA, probs = c(0.25), na.rm = TRUE),

Q3 = quantile(días_SONDA, probs = c(0.75), na.rm = TRUE))

summary_3

wilcox.test(días_SONDA ~ COVID, data=BASE_3)

CrossTable(BASE_3$COVID, BASE_3$UROCULTIVO_POSITIVO)

chisq.test(BASE_3$COVID, BASE_3$UROCULTIVO_POSITIVO)

CrossTable(BASE_3$COVID, BASE_3$ISTU_AC)

chisq.test(BASE_3$COVID, BASE_3$ISTU_AC)

CrossTable(BASE_3$COVID, BASE_3$USO_OROTRAQUEAL_TRAQUEOSTOMIA)

chisq.test(BASE_3$COVID, BASE_3$USO_OROTRAQUEAL_TRAQUEOSTOMIA)

BASE_3$días_INTUBACIÓN <- time_length(BASE_3$FECHA_FINAL_OROTRAQUEAL_TRAQUEOSTOMIA - BASE_3$FECHA_OROTRAQUEAL_TRAQUEOSTOMIA, unit="days")

summary_4 <- BASE_3 %>%

group_by(COVID) %>%

dplyr::summarize(mean = mean(días_INTUBACIÓN, na.rm = TRUE),

sd = sd(días_INTUBACIÓN, na.rm = TRUE),

median = median(días_INTUBACIÓN, na.rm = TRUE),

Q1 = quantile(días_INTUBACIÓN, probs = c(0.25), na.rm = TRUE),

Q3 = quantile(días_INTUBACIÓN, probs = c(0.75), na.rm = TRUE))

summary_4

wilcox.test(días_INTUBACIÓN ~ COVID, data=BASE_3)

CrossTable(BASE_3$COVID, BASE_3$CULTIVO_NEUMONIA_POSITIVAS)

chisq.test(BASE_3$COVID, BASE_3$CULTIVO_NEUMONIA_POSITIVAS)

CrossTable(BASE_3$COVID, BASE_3$NEU)

chisq.test(BASE_3$COVID, BASE_3$NEU)

CrossTable(BASE_3$COVID, BASE_3$IAAS_GRAM_POS)

chisq.test(BASE_3$COVID, BASE_3$IAAS_GRAM_POS)

CrossTable(BASE_3$COVID, BASE_3$IAAS_GRAM_NEG)

chisq.test(BASE_3$COVID, BASE_3$IAAS_GRAM_NEG)

CrossTable(BASE_3$COVID, BASE_3$IAAS_ATIPICOS)

chisq.test(BASE_3$COVID, BASE_3$IAAS_ATIPICOS)

CrossTable(BASE_3$COVID, BASE_3$IAAS_HONGOS)

chisq.test(BASE_3$COVID, BASE_3$IAAS_HONGOS)

#Analisis frecuencia de aislamientos general

NEW_BASE_1 <- BASE %>%

group_by(COVID) %>%

summarise(S._aureus = sum(S._aureus == "1")

,E._coli = sum(E._coli == "1")

,Candida_spp = sum(Candida_spp == "1")

,K._pneumoniae = sum(K._pneumoniae == "1")

,E._cloacae = sum(E._cloacae == "1")

,S._malthophila = sum(S._malthophila == "1")

,S._Epidermidis = sum(S._Epidermidis == "1")

,E._faecalis = sum(E._faecalis == "1")

,P._aeruginosa = sum(P._aeruginosa == "1")

,S._marcensens = sum(S._marcensens == "1")

,P._mirabilis = sum(P._mirabilis == "1")

,E._aerogenes = sum(E._aerogenes == "1")

,B._cepacia = sum(B._cepacia == "1")

,K._oxytoca = sum(K._oxytoca == "1")

,K._variicola = sum(K._variicola == "1")

,S._agalactiae = sum(S._agalactiae == "1")

,S._hominis = sum(S._hominis == "1")

,S._pneumoniae = sum(S._pneumoniae == "1")

,C._koseri = sum(C._koseri == "1")

,A._fumigatus = sum(A._fumigatus == "1")

,M._catarrhalis = sum(M._catarrhalis == "1")

)

View(NEW_BASE_1)

NEW_BASE_1 <- NEW_BASE_1 %>%

pivot_longer(cols = -COVID, names_to = "PATOGENO", values_to = "AISLAMIENTOS")

NEW_BASE_1.1 <- subset(NEW_BASE_1, COVID == "SI")

sum1.1 <- with(NEW_BASE_1.1, sum(AISLAMIENTOS))

NEW_BASE_1.1$porcentaje <- NEW_BASE_1.1$AISLAMIENTOS / sum1.1

NEW_BASE_1.2 <- subset(NEW_BASE_1, COVID == "NO")

sum1.2 <- with(NEW_BASE_1.2, sum(AISLAMIENTOS))

NEW_BASE_1.2$porcentaje <- NEW_BASE_1.2$AISLAMIENTOS / sum1.2

NEW_BASE_1 <- rbind(NEW_BASE_1.2, NEW_BASE_1.1)

NEW_BASE_1$porcentaje <- NEW_BASE_1$porcentaje * 100

Fig_A <- NEW_BASE_1 %>%

ggplot(aes(x = PATOGENO, y = porcentaje, fill = COVID)) +

geom_col(position = "dodge") +

coord_flip() +

theme_classic() +

scale_fill_manual(values=c("#E7B800","#00AFBB"), name = "COVID-19

diagnose", labels=c("NO", "YES")) +labs(title="Percentage of Identified Pathogens in Invasive Medical Devices in ICU" , x ="Phatogens", y = "Identification percentage (%)") + scale_x_discrete(labels=c('A. fumigatus', 'B. cepacia', 'C. koseri', 'Candida spp','E. aerogenes', 'E. cloacae', 'E. coli', 'E. faecalis','K. oxytoca', 'K. pneumoniae', 'K. variicola', 'M. catarrhalis', 'P. aeruginosa','P. mirabilis', 'S. agalactiae', 'S. aureus', 'S. Epidermidis', 'S. hominis', 'S. malthophila', 'S. marcensens', 'S. pneumoniae'))

Fig_A <- Fig_A + theme(axis.text=element_text(size=8), axis.title = element_text(size = 8), title = element_text(size = 8), legend.text = element_text(size = 6))

#calcular frecuencia de patogenos por dispositivo

NEW_BASE_2 <- BASE %>%

group_by(COVID) %>%

summarise(B_cepacia = sum(PATOGENO_HEMOCULTIVO == "B. cepacia")

,Candida_spp = sum(PATOGENO_HEMOCULTIVO == "Candida spp")

,E_coli = sum(PATOGENO_HEMOCULTIVO == "E. coli")

,S_hominis = sum(PATOGENO_HEMOCULTIVO == "S. hominis")

,K_pneumoniae_KPC= sum(PATOGENO_HEMOCULTIVO == "K. pneumoniae (KPC)")

,P_aeruginosa = sum(PATOGENO_HEMOCULTIVO == "P. aeruginosa")

,E_faecalis = sum(PATOGENO_HEMOCULTIVO == "E. faecalis")

,E_cloacae = sum(PATOGENO_HEMOCULTIVO == "S. epidermidis")

,E_cloacae = sum(PATOGENO_HEMOCULTIVO == "E. cloacae")

,S_epidermidis = sum(PATOGENO_HEMOCULTIVO == "S. epidermidis")

,S_agalactiae= sum(PATOGENO_HEMOCULTIVO == "S. agalactiae")

,P_mirabilis = sum(PATOGENO_HEMOCULTIVO == "P. mirabilis")

,K_pneumoniae = sum(PATOGENO_HEMOCULTIVO == "K. pneumoniae")

,S_argentus = sum(PATOGENO_HEMOCULTIVO == "S. argentus")

,K_oxytoca = sum(PATOGENO_HEMOCULTIVO == "K. oxytoca")

,SAMS = sum(PATOGENO_HEMOCULTIVO == "SAMS")

,SAMR = sum(PATOGENO_HEMOCULTIVO == "SAMR")

)

View(NEW_BASE_2)

NEW_BASE_2 <- NEW_BASE_2 %>%

pivot_longer(cols = -COVID, names_to = "PATOGENO", values_to = "AISLAMIENTOS")

NEW_BASE_2.1 <- subset(NEW_BASE_2, COVID == "SI")

sum2.1 <- with(NEW_BASE_2.1, sum(AISLAMIENTOS))

NEW_BASE_2.1$porcentaje <- NEW_BASE_2.1$AISLAMIENTOS / sum2.1

NEW_BASE_2.2 <- subset(NEW_BASE_2, COVID == "NO")

sum2.2 <- with(NEW_BASE_2.2, sum(AISLAMIENTOS))

NEW_BASE_2.2$porcentaje <- NEW_BASE_2.2$AISLAMIENTOS / sum2.2

NEW_BASE_2 <- rbind(NEW_BASE_2.2, NEW_BASE_2.1)

NEW_BASE_2$porcentaje <- NEW_BASE_2$porcentaje * 100

Fig_B <- NEW_BASE_2 %>%

ggplot( aes(x = PATOGENO, y = porcentaje, fill = COVID, )) +

geom_col(position = "dodge") +

coord_flip() +

theme_classic() +

scale_fill_manual(values=c("#E7B800","#00AFBB"), name = "COVID-19

diagnose", labels=c("NO", "YES")) +labs(title="Percentage of Main Pathogens Isolated in Central Venous Catheter in ICU") + xlab ("") + ylab ("Insolated percentage (%)") + scale_x_discrete(labels=c('B. cepacia', 'Candida spp', 'E. cloacae', 'E. coli', 'E. faecalis','K. oxytoca', 'K. pneumoniae', 'K. pneumoniae (KPC)', 'P. aeruginosa','P. mirabilis', 'S. agalactiae', 'S. argentus', 'S. Epidermidis', 'S. hominis', 'MRSA', 'MSSA'))

Fig_B <- Fig_B + theme(axis.text=element_text(size=8), axis.title = element_text(size = 8), title = element_text(size = 8), legend.text = element_text(size = 6))

NEW_BASE_3 <- BASE %>%

group_by(COVID) %>%

summarise(Candida_spp = sum(PATOGENO_UROCULTIVO == "Candida spp")

,E_coli = sum(PATOGENO_UROCULTIVO == "E. coli")

,K_pneumoniae_KPC= sum(PATOGENO_UROCULTIVO == "K. pneumoniae (KPC)")

,E_faecalis = sum(PATOGENO_UROCULTIVO == "E. faecalis")

,E_cloacae = sum(PATOGENO_UROCULTIVO == "E. cloacae")

,K_pneumoniae = sum(PATOGENO_UROCULTIVO == "K. pneumoniae")

)

View(NEW_BASE_3)

NEW_BASE_3 <- NEW_BASE_3 %>%

pivot_longer(cols = -COVID, names_to = "PATOGENO", values_to = "AISLAMIENTOS")

NEW_BASE_3.1 <- subset(NEW_BASE_3, COVID == "SI")

sum3.1 <- with(NEW_BASE_3.1, sum(AISLAMIENTOS))

NEW_BASE_3.1$porcentaje <- NEW_BASE_3.1$AISLAMIENTOS / sum3.1

NEW_BASE_3.2 <- subset(NEW_BASE_3, COVID == "NO")

sum3.2 <- with(NEW_BASE_3.2, sum(AISLAMIENTOS))

NEW_BASE_3.2$porcentaje <- NEW_BASE_3.2$AISLAMIENTOS / sum3.2

NEW_BASE_3 <- rbind(NEW_BASE_3.2, NEW_BASE_3.1)

NEW_BASE_3$porcentaje <- NEW_BASE_3$porcentaje * 100

Fig_C <- NEW_BASE_3 %>%

ggplot( aes(x = PATOGENO, y = porcentaje, fill = COVID, )) +

geom_col(position = "dodge") +

coord_flip() +

theme_classic() +

scale_fill_manual(values=c("#E7B800","#00AFBB"), name = "COVID-19

diagnose", labels=c("NO", "YES")) +labs(title="Percentage of Main Pathogens Isolated in Urinary Catheter in ICU", x ="Phatogens", y = "Insolated percentage (%)") + scale_x_discrete(labels=c('Candida spp', 'E. cloacae', 'E. coli', 'E. faecalis', 'K. pneumoniae', 'K. pneumoniae (KPC)'))

Fig_C <- Fig_C + theme(axis.text=element_text(size=8), axis.title = element_text(size = 8), title = element_text(size = 8), legend.text = element_text(size = 6))

NEW_BASE_4 <- BASE %>%

group_by(COVID) %>%

summarise(B_cepacia = sum(PATOGENO_SECRECIONES == "B. cepacia")

,Candida_spp = sum(PATOGENO_SECRECIONES == "Candida spp")

,E_coli = sum(PATOGENO_SECRECIONES == "E. coli")

,K_pneumoniae_KPC= sum(PATOGENO_SECRECIONES == "K. pneumoniae (KPC)")

,P_aeruginosa = sum(PATOGENO_SECRECIONES == "P. aeruginosa")

,E_cloacae = sum(PATOGENO_SECRECIONES == "E. cloacae")

,K_pneumoniae = sum(PATOGENO_SECRECIONES == "K. pneumoniae")

,K_oxytoca = sum(PATOGENO_SECRECIONES == "K. oxytoca")

,SAMS = sum(PATOGENO_SECRECIONES == "SAMS")

,SAMR = sum(PATOGENO_SECRECIONES == "SAMR")

)

View(NEW_BASE_4)

NEW_BASE_4 <- NEW_BASE_4 %>%

pivot_longer(cols = -COVID, names_to = "PATOGENO", values_to = "AISLAMIENTOS")

NEW_BASE_4.1 <- subset(NEW_BASE_4, COVID == "SI")

sum4.1 <- with(NEW_BASE_4.1, sum(AISLAMIENTOS))

NEW_BASE_4.1$porcentaje <- NEW_BASE_4.1$AISLAMIENTOS / sum4.1

NEW_BASE_4.2 <- subset(NEW_BASE_4, COVID == "NO")

sum4.2 <- with(NEW_BASE_4.2, sum(AISLAMIENTOS))

NEW_BASE_4.2$porcentaje <- NEW_BASE_4.2$AISLAMIENTOS / sum4.2

NEW_BASE_4 <- rbind(NEW_BASE_4.2, NEW_BASE_4.1)

NEW_BASE_4$porcentaje <- NEW_BASE_4$porcentaje * 100

Fig_D <- NEW_BASE_4 %>%

ggplot( aes(x = PATOGENO, y = porcentaje, fill = COVID, )) +

geom_col(position = "dodge") +

coord_flip() +

theme_classic() +

scale_fill_manual(values=c("#E7B800","#00AFBB"), name = "COVID-19

diagnose", labels=c("NO", "YES")) +labs(title="Main Pathogens Isolated in Invasive Ventilation in ICU", x ="", y = "Insolated percentage (%)") + scale_x_discrete(labels=c('B. cepacia','Candida spp', 'E. cloacae', 'E. coli','K. oxytoca', 'K. pneumoniae', 'K. pneumoniae (KPC)','P. aeruginosa', 'MRSA', 'MSSA'))

Fig_D <- Fig_D + theme(axis.text=element_text(size=8), axis.title = element_text(size = 8), title = element_text(size = 8), legend.text = element_text(size = 6))

ggarrange(Fig_A, Fig_B, Fig_C, Fig_D,

ncol = 2, nrow = 2)

# análisis inferencial

CrossTable(BASE$COVID, BASE$E._coli)

chisq.test(BASE$COVID, BASE$E._coli)

CrossTable(BASE$COVID, BASE$S._aureus)

chisq.test(BASE$COVID, BASE$S._aureus)

CrossTable(BASE$COVID, BASE$S._pneumoniae)

chisq.test(BASE$COVID, BASE$S._pneumoniae)

CrossTable(BASE$COVID, BASE$S._marcensens)

chisq.test(BASE$COVID, BASE$S._marcensens)

CrossTable(BASE$COVID, BASE$S._malthophila)

chisq.test(BASE$COVID, BASE$S._malthophila)

CrossTable(BASE$COVID, BASE$S._hominis)

chisq.test(BASE$COVID, BASE$S._hominis)

CrossTable(BASE$COVID, BASE$S._Epidermidis)

chisq.test(BASE$COVID, BASE$S._Epidermidis)

CrossTable(BASE$COVID, BASE$S._agalactiae)

chisq.test(BASE$COVID, BASE$S._agalactiae)

CrossTable(BASE$COVID, BASE$P._mirabilis)

chisq.test(BASE$COVID, BASE$P._mirabilis)

CrossTable(BASE$COVID, BASE$P._aeruginosa)

chisq.test(BASE$COVID, BASE$P._aeruginosa)

CrossTable(BASE$COVID, BASE$M._catarrhalis)

chisq.test(BASE$COVID, BASE$M._catarrhalis)

CrossTable(BASE$COVID, BASE$K._variicola)

chisq.test(BASE$COVID, BASE$K._variicola)

CrossTable(BASE$COVID, BASE$K._pneumoniae)

chisq.test(BASE$COVID, BASE$K._pneumoniae)

CrossTable(BASE$COVID, BASE$K._oxytoca)

chisq.test(BASE$COVID, BASE$K._oxytoca)

CrossTable(BASE$COVID, BASE$E._faecalis)

chisq.test(BASE$COVID, BASE$E._faecalis)

CrossTable(BASE$COVID, BASE$E._cloacae)

chisq.test(BASE$COVID, BASE$E._cloacae)

CrossTable(BASE$COVID, BASE$E._aerogenes)

chisq.test(BASE$COVID, BASE$E._aerogenes)

CrossTable(BASE$COVID, BASE$Candida_spp)

chisq.test(BASE$COVID, BASE$Candida_spp)

CrossTable(BASE$COVID, BASE$C._koseri)

chisq.test(BASE$COVID, BASE$C._koseri)

CrossTable(BASE$COVID, BASE$B._cepacia)

chisq.test(BASE$COVID, BASE$B._cepacia)

CrossTable(BASE$COVID, BASE$A._fumigatus)

chisq.test(BASE$COVID, BASE$A._fumigatus)

table_1 <- table(BASE$E._coli, BASE$COVID)

riskratio(t(table_1))

table_2 <- table(BASE$S._aureus, BASE$COVID)

riskratio(t(table_2))

table_3 <- table(BASE$K._pneumoniae, BASE$COVID)

riskratio(t(table_3))

table_4 <- table(BASE$E._faecalis, BASE$COVID)

riskratio(t(table_4))

table_5 <- table(BASE$Candida_spp, BASE$COVID)

riskratio(t(table_5))

table_6 <- table(BASE$S._pneumoniae, BASE$COVID)

riskratio(t(table_6))

table_7 <- table(BASE$S._marcensens, BASE$COVID)

riskratio(t(table_7))

table_8 <- table(BASE$S._malthophila, BASE$COVID)

riskratio(t(table_8))

table_9 <- table(BASE$S._hominis, BASE$COVID)

riskratio(t(table_9))

table_10 <- table(BASE$S._Epidermidis, BASE$COVID)

riskratio(t(table_10))

table_11 <- table(BASE$S._agalactiae, BASE$COVID)

riskratio(t(table_11))

table_12 <- table(BASE$P._mirabilis, BASE$COVID)

riskratio(t(table_12))

table_13 <- table(BASE$P._aeruginosa, BASE$COVID)

riskratio(t(table_13))

table_14 <- table(BASE$M._catarrhalis, BASE$COVID)

riskratio(t(table_14))

table_15 <- table(BASE$K._variicola, BASE$COVID)

riskratio(t(table_15))

table_16 <- table(BASE$K._oxytoca, BASE$COVID)

riskratio(t(table_16))

table_17 <- table(BASE$E._cloacae, BASE$COVID)

riskratio(t(table_17))

table_18 <- table(BASE$E._aerogenes, BASE$COVID)

riskratio(t(table_18))

table_19 <- table(BASE$C._koseri, BASE$COVID)

riskratio(t(table_19))

table_20 <- table(BASE$B._cepacia, BASE$COVID)

riskratio(t(table_20))

table_21 <- table(BASE$A._fumigatus, BASE$COVID)

riskratio(t(table_21))

#Análisis general tiempo al evento

BASE$Tiempo_IAAS <- time_length(BASE$FECHA_SINTOMAS_INFECCION - BASE$FECHA_INGRESO_UCI, unit="days")

View(BASE)

BASE$Tiempo_IAAS[is.na(BASE$Tiempo_IAAS)] <- 100

BASE['IAAS'][BASE['IAAS'] == 'NO'] <- '0'

BASE['IAAS'][BASE['IAAS'] == 'SI'] <- '1'

BASE$IAAS <- as.numeric(as.character(BASE$IAAS))

#sobrevida general

fit_1 <- survfit(Surv(DÍAS_UCI, DESENLACE) ~ COVID, data=BASE)

print(fit_1)

ggsurvplot(fit_1,

pval = TRUE,

conf.int = TRUE,

risk.table.col = "strata", # Change risk table color by groups

ggtheme = theme_bw(), # Change ggplot2 theme

palette = c("#E7B800","#00AFBB"),

xlim = c(0, 30))

fit_2 <- survfit(Surv(DÍAS_UCI, DESENLACE) ~ COVID, data=BASE_2)

print(fit_2)

ggsurvplot(fit_2,

pval = TRUE,

conf.int = TRUE,

risk.table.col = "strata", # Change risk table color by groups

ggtheme = theme_bw(), # Change ggplot2 theme

palette = c("#E7B800","#00AFBB"),

xlim = c(0, 30))

fit_3 <- survfit(Surv(DÍAS_UCI, DESENLACE) ~ COVID, data=BASE_3)

print(fit_3)

ggsurvplot(fit_3,

pval = TRUE,

conf.int = TRUE,

risk.table.col = "strata", # Change risk table color by groups

ggtheme = theme_bw(), # Change ggplot2 theme

palette = c("#E7B800","#00AFBB"),

xlim = c(0, 30))

# IAAS tiempo al evento general

splots_1 <- list()

BASE$Tiempo_IAAS <- time_length(BASE$FECHA_SINTOMAS_INFECCION - BASE$FECHA_INGRESO_UCI, unit="days")

View(BASE)

BASE$Tiempo_IAAS[is.na(BASE$Tiempo_IAAS)] <- 40

BASE$Tiempo_VENTILACION <- time_length(BASE$FECHA_FINAL_OROTRAQUEAL_TRAQUEOSTOMIA - BASE$FECHA_OROTRAQUEAL_TRAQUEOSTOMIA, unit="days")

View(BASE)

BASE['IAAS'][BASE['IAAS'] == 'NO'] <- '0'

BASE['IAAS'][BASE['IAAS'] == 'SI'] <- '1'

BASE$IAAS <- as.numeric(as.character(BASE$IAAS))

fit_4 <- survfit(Surv(Tiempo_IAAS, IAAS) ~ COVID, data = BASE)

print(fit_4)

splots_1[[1]] <- ggsurvplot(fit_4,

pval = TRUE,

pval.coord = c(0, 0.65),

pval.size = c(3),

conf.int = TRUE,

risk.table.col = "strata", # Change risk table color by groups

ggtheme = theme_classic(), # Change ggplot2 theme

palette = c("#E7B800","#00AFBB"),

fun = "event",

legend = "none",

legend.title = "Covid-19 diagnose",

legend.labs = c("No", "Yes"),

xlim = c(0, 30), ylim = c(0, 0.7)) + ggtitle("A Total Cohort") + xlab("") + ylab("Cumulative HAIs incidence")

splots_1[[1]] <- ggpar(splots_1[[1]],

font.main = c(12, "bold"),

font.y = c(12, "bold"),

font.tickslab = c(10, "bold"))

fit_5 <- survfit(Surv(Tiempo_IAAS, IAAS) ~ COVID, data = BASE_2)

print(fit_5)

splots_1[[2]] <- ggsurvplot(fit_5,

pval = TRUE,

pval.coord = c(0, 0.65),

pval.size = c(3),

conf.int = TRUE,

risk.table.col = "strata", # Change risk table color by groups

ggtheme = theme_classic(), # Change ggplot2 theme

palette = c("#E7B800","#00AFBB"),

fun = "event",

legend = c(0.15, 0.75),

legend.title = "Covid-19 diagnose",

legend.labs = c("No", "Yes"),

xlim = c(0, 30), ylim = c(0, 0.7)) + ggtitle("B Cohort adjusted by days in ICU") + xlab("Days") + ylab("")

splots_1[[2]] <- ggpar(splots_1[[2]],

font.main = c(12, "bold"),

font.x = c(12, "bold"),

font.subtitle = c(2, "bold"),

font.legend = c(8, "bold"),

font.tickslab = c(10, "bold"))

fit_6 <- survfit(Surv(Tiempo_IAAS, IAAS) ~ COVID, data = BASE_3)

print(fit_6)

splots_1[[3]] <- ggsurvplot(fit_6,

pval = TRUE,

pval.coord = c(0, 0.65),

pval.size = c(3),

conf.int = TRUE,

risk.table.col = "strata", # Change risk table color by groups

ggtheme = theme_classic(), # Change ggplot2 theme

palette = c("#E7B800","#00AFBB"),

fun = "event",

legend = "none",

legend.title = "Covid-19 diagnose",

legend.labs = c("No", "Yes"),

xlim = c(0, 30), ylim = c(0, 0.7)) + ggtitle("C Cohort adjusted for ventilation days") + xlab("") + ylab("")

splots_1[[3]] <- ggpar(splots_1[[3]],

font.main = c(12, "bold"),

font.tickslab = c(10, "bold"))

plot_1 <- arrange_ggsurvplots(splots_1, print = TRUE, ncol = 3, nrow =1)

#Tiempo al evento por tipo de patogeno

splots_2 <- list()

fit_7 <- survfit(Surv(Tiempo_gram_pos, IAAS_GRAM_POS) ~ COVID, data = BASE)

print(fit_7)

splots_2[[1]] <- ggsurvplot(fit_7,

pval = TRUE,

pval.coord = c(0, 0.35),

pval.size = c(3),

conf.int = TRUE,

risk.table.col = "strata", # Change risk table color by groups

ggtheme = theme_classic(), # Change ggplot2 theme

palette = c("#E7B800","#00AFBB"),

fun = "event",

legend = "none",

legend.title = "Covid-19 diagnose",

legend.labs = c("No", "Yes"),

xlim = c(0, 30), ylim = c(0, 0.4)) + ggtitle("A Total Cohort") + xlab("") + ylab("Cumulative Gram positive

HAIs incidence")

splots_2[[1]] <- ggpar(splots_2[[1]],

font.main = c(12, "bold"),

font.y = c(8, "bold"),

font.tickslab = c(8, "bold"))

fit_8 <- survfit(Surv(FECHA_GRAM_NEG, IAAS_GRAM_NEG) ~ COVID, data = BASE)

print(fit_8)

splots_2[[2]] <- ggsurvplot(fit_8,

pval = TRUE,

pval.coord = c(0, 0.35),

pval.size = c(3),

conf.int = TRUE,

risk.table.col = "strata", # Change risk table color by groups

ggtheme = theme_classic(), # Change ggplot2 theme

palette = c("#E7B800","#00AFBB"),

fun = "event",

legend = "none",

legend.title = "Covid-19 diagnose",

legend.labs = c("No", "Yes"),

xlim = c(0, 30), ylim = c(0, 0.4)) + ggtitle("B") + xlab("") + ylab("Cumulative Gram negative

HAIs incidence")

splots_2[[2]] <- ggpar(splots_2[[2]],

font.main = c(12, "bold"),

font.y = c(8, "bold"),

font.tickslab = c(8, "bold"))

fit_9 <- survfit(Surv(FECHA_HONGOS, IAAS_HONGOS) ~ COVID, data = BASE)

print(fit_9)

splots_2[[3]] <- ggsurvplot(fit_9,

pval = TRUE,

pval.coord = c(0, 0.35),

pval.size = c(3),

conf.int = TRUE,

risk.table.col = "strata", # Change risk table color by groups

ggtheme = theme_classic(), # Change ggplot2 theme

palette = c("#E7B800","#00AFBB"),

fun = "event",

legend = "none",

legend.title = "Covid-19 diagnose",

legend.labs = c("No", "Yes"),

xlim = c(0, 30), ylim = c(0, 0.4)) + ggtitle("C") + xlab("") + ylab("Cumulative Fungie

HAIs incidence")

splots_2[[3]] <- ggpar(splots_2[[3]],

font.main = c(12, "bold"),

font.y = c(8, "bold"),

font.tickslab = c(8, "bold"))

fit_10 <- survfit(Surv(FECHA_ATIPICOS, IAAS_ATIPICOS) ~ COVID, data = BASE)

print(fit_10)

splots_2[[4]] <- ggsurvplot(fit_10,

pval = TRUE,

pval.coord = c(0, 0.35),

pval.size = c(3),

conf.int = TRUE,

risk.table.col = "strata", # Change risk table color by groups

ggtheme = theme_classic(), # Change ggplot2 theme

palette = c("#E7B800","#00AFBB"),

fun = "event",

legend = "none",

legend.title = "Covid-19 diagnose",

legend.labs = c("No", "Yes"),

xlim = c(0, 30), ylim = c(0, 0.4)) + ggtitle("D") + xlab("") + ylab("Cumulative Atipic

HAIs incidence")

splots_2[[4]] <- ggpar(splots_2[[4]],

font.main = c(12, "bold"),

font.y = c(8, "bold"),

font.tickslab = c(8, "bold"))

fit_11 <- survfit(Surv(Tiempo_gram_pos, IAAS_GRAM_POS) ~ COVID, data = BASE_2)

print(fit_11)

splots_2[[5]] <- ggsurvplot(fit_11,

pval = TRUE,

pval.coord = c(0, 0.35),

pval.size = c(3),

conf.int = TRUE,

risk.table.col = "strata", # Change risk table color by groups

ggtheme = theme_classic(), # Change ggplot2 theme

palette = c("#E7B800","#00AFBB"),

fun = "event",

legend = "none",

legend.title = "Covid-19 diagnose",

legend.labs = c("No", "Yes"),

xlim = c(0, 30), ylim = c(0, 0.4)) + ggtitle("E Cohort adjusted by days in ICU") + xlab("") + ylab("")

splots_2[[5]] <- ggpar(splots_2[[5]],

font.main = c(12, "bold"),

font.tickslab = c(8, "bold"))

fit_12 <- survfit(Surv(FECHA_GRAM_NEG, IAAS_GRAM_NEG) ~ COVID, data = BASE_2)

print(fit_12)

splots_2[[6]] <- ggsurvplot(fit_12,

pval = TRUE,

pval.coord = c(0, 0.35),

pval.size = c(3),

conf.int = TRUE,

risk.table.col = "strata", # Change risk table color by groups

ggtheme = theme_classic(), # Change ggplot2 theme

palette = c("#E7B800","#00AFBB"),

fun = "event",

legend = "none",

legend.title = "Covid-19 diagnose",

legend.labs = c("No", "Yes"),

xlim = c(0, 30), ylim = c(0, 0.4)) + ggtitle("F") + xlab("") + ylab("")

splots_2[[6]] <- ggpar(splots_2[[6]],

font.main = c(12, "bold"),

font.tickslab = c(8, "bold"))

fit_13 <- survfit(Surv(FECHA_HONGOS, IAAS_HONGOS) ~ COVID, data = BASE_2)

print(fit_13)

splots_2[[7]] <- ggsurvplot(fit_13,

pval = TRUE,

pval.coord = c(0, 0.35),

pval.size = c(3),

conf.int = TRUE,

risk.table.col = "strata", # Change risk table color by groups

ggtheme = theme_classic(), # Change ggplot2 theme

palette = c("#E7B800","#00AFBB"),

fun = "event",

legend = "none",

legend.title = "Covid-19 diagnose",

legend.labs = c("No", "Yes"),

xlim = c(0, 30), ylim = c(0, 0.4)) + ggtitle("G") + xlab("") + ylab("")

splots_2[[7]] <- ggpar(splots_2[[7]],

font.main = c(12, "bold"),

font.tickslab = c(8, "bold"))

fit_14 <- survfit(Surv(FECHA_ATIPICOS, IAAS_ATIPICOS) ~ COVID, data = BASE_2)

print(fit_14)

splots_2[[8]] <- ggsurvplot(fit_14,

pval = TRUE,

pval.coord = c(0, 0.35),

pval.size = c(3),

conf.int = TRUE,

risk.table.col = "strata", # Change risk table color by groups

ggtheme = theme_classic(), # Change ggplot2 theme

palette = c("#E7B800","#00AFBB"),

fun = "event",

legend = "none",

legend.title = "Covid-19 diagnose",

legend.labs = c("No", "Yes"),

xlim = c(0, 30), ylim = c(0, 0.4)) + ggtitle("H") + xlab("Days") + ylab("")

splots_2[[8]] <- ggpar(splots_2[[8]],

font.main = c(12, "bold"),

font.x = c(8, "bold"),

font.tickslab = c(8, "bold"))

fit_15 <- survfit(Surv(Tiempo_gram_pos, IAAS_GRAM_POS) ~ COVID, data = BASE_3)

print(fit_15)

splots_2[[9]] <- ggsurvplot(fit_15,

pval = TRUE,

pval.coord = c(0, 0.35),

pval.size = c(3),

conf.int = TRUE,

risk.table.col = "strata", # Change risk table color by groups

ggtheme = theme_classic(), # Change ggplot2 theme

palette = c("#E7B800","#00AFBB"),

fun = "event",

legend = "none",

legend.title = "Covid-19 diagnose",

legend.labs = c("No", "Yes"),

xlim = c(0, 30), ylim = c(0, 0.4)) + ggtitle("I Cohort adjusted for ventilation days") + xlab("") + ylab("")

splots_2[[9]] <- ggpar(splots_2[[9]],

font.main = c(12, "bold"),

font.tickslab = c(8, "bold"))

fit_16 <- survfit(Surv(FECHA_GRAM_NEG, IAAS_GRAM_NEG) ~ COVID, data = BASE_3)

print(fit_16)

splots_2[[10]] <- ggsurvplot(fit_16,

pval = TRUE,

pval.coord = c(0, 0.35),

pval.size = c(3),

conf.int = TRUE,

risk.table.col = "strata", # Change risk table color by groups

ggtheme = theme_classic(), # Change ggplot2 theme

palette = c("#E7B800","#00AFBB"),

fun = "event",

legend = "none",

legend.title = "Covid-19 diagnose",

legend.labs = c("No", "Yes"),

xlim = c(0, 30), ylim = c(0, 0.4)) + ggtitle("J") + xlab("") + ylab("")

splots_2[[10]] <- ggpar(splots_2[[10]],

font.main = c(12, "bold"),

font.tickslab = c(8, "bold"))

fit_17 <- survfit(Surv(FECHA_HONGOS, IAAS_HONGOS) ~ COVID, data = BASE_3)

print(fit_17)

splots_2[[11]] <- ggsurvplot(fit_17,

pval = TRUE,

pval.coord = c(0, 0.35),

pval.size = c(3),

conf.int = TRUE,

risk.table.col = "strata", # Change risk table color by groups

ggtheme = theme_classic(), # Change ggplot2 theme

palette = c("#E7B800","#00AFBB"),

fun = "event",

legend = "none",

legend.title = "Covid-19 diagnose",

legend.labs = c("No", "Yes"),

xlim = c(0, 30), ylim = c(0, 0.4)) + ggtitle("K") + xlab("") + ylab("")

splots_2[[11]] <- ggpar(splots_2[[11]],

font.main = c(12, "bold"),

font.tickslab = c(8, "bold"))

fit_18 <- survfit(Surv(FECHA_ATIPICOS, IAAS_ATIPICOS) ~ COVID, data = BASE_3)

print(fit_18)

splots_2[[12]] <- ggsurvplot(fit_18,

pval = TRUE,

pval.coord = c(0, 0.35),

pval.size = c(3),

conf.int = TRUE,

risk.table.col = "strata", # Change risk table color by groups

ggtheme = theme_classic(), # Change ggplot2 theme

palette = c("#E7B800","#00AFBB"),

fun = "event",

legend = c(0.7, 0.71),

legend.title = "Covid-19 diagnose",

legend.labs = c("No", "Yes"),

xlim = c(0, 30), ylim = c(0, 0.4)) + ggtitle("L") + xlab("") + ylab("")

splots_2[[12]] <- ggpar(splots_2[[12]],

font.main = c(12, "bold"),

font.legend = c(8, "bold"),

font.tickslab = c(8, "bold"))

plot_2 <- arrange_ggsurvplots(splots_2, print = TRUE, ncol = 3, nrow =4)

#Tiempo al evento por dispositivo

splots_3 <- list()

fit_19 <- survfit(Surv(Tiempo_ITS_AC, ITS_AC) ~ COVID, data = BASE)

print(fit_19)

splots_3[[1]] <- ggsurvplot(fit_19,

pval = TRUE,

pval.coord = c(0, 0.35),

pval.size = c(3),

conf.int = TRUE,

risk.table.col = "strata", # Change risk table color by groups

ggtheme = theme_classic(), # Change ggplot2 theme

palette = c("#E7B800","#00AFBB"),

fun = "event",

legend = "none",

legend.title = "Covid-19 diagnose",

legend.labs = c("No", "Yes"),

xlim = c(0, 30), ylim = c(0, 0.55)) + ggtitle("A Total Cohort") + xlab("") + ylab("Cumulative ITS AC incidence")

splots_3[[1]] <- ggpar(splots_3[[1]],

font.main = c(12, "bold"),

font.legend = c(8, "bold"),

font.tickslab = c(8, "bold"))

fit_20 <- survfit(Surv(Tiempo_ISTU_AC, ISTU_AC) ~ COVID, data = BASE)

print(fit_20)

splots_3[[2]] <- ggsurvplot(fit_20,

pval = TRUE,

pval.coord = c(0, 0.35),

pval.size = c(3),

conf.int = TRUE,

risk.table.col = "strata", # Change risk table color by groups

ggtheme = theme_classic(), # Change ggplot2 theme

palette = c("#E7B800","#00AFBB"),

fun = "event",

legend = "none",

legend.title = "Covid-19 diagnose",

legend.labs = c("No", "Yes"),

xlim = c(0, 30), ylim = c(0, 0.55)) + ggtitle("B") + xlab("") + ylab("Cumulative ISTU incidence")

splots_3[[2]] <- ggpar(splots_3[[2]],

font.main = c(12, "bold"),

font.legend = c(8, "bold"),

font.tickslab = c(8, "bold"))

fit_21 <- survfit(Surv(Tiempo_NEU, NEU) ~ COVID, data = BASE)

print(fit_21)

splots_3[[3]] <- ggsurvplot(fit_21,

pval = TRUE,

pval.coord = c(0, 0.35),

pval.size = c(3),

conf.int = TRUE,

risk.table.col = "strata", # Change risk table color by groups

ggtheme = theme_classic(), # Change ggplot2 theme

palette = c("#E7B800","#00AFBB"),

fun = "event",

legend = "none",

legend.title = "Covid-19 diagnose",

legend.labs = c("No", "Yes"),

xlim = c(0, 30), ylim = c(0, 0.55)) + ggtitle("C") + xlab("") + ylab("Cumulative VAP incidence")

splots_3[[3]] <- ggpar(splots_3[[3]],

font.main = c(12, "bold"),

font.legend = c(8, "bold"),

font.tickslab = c(8, "bold"))

fit_22 <- survfit(Surv(Tiempo_ITS_AC, ITS_AC) ~ COVID, data = BASE_2)

print(fit_22)

splots_3[[4]] <- ggsurvplot(fit_22,

pval = TRUE,

pval.coord = c(0, 0.35),

pval.size = c(3),

conf.int = TRUE,

risk.table.col = "strata", # Change risk table color by groups

ggtheme = theme_classic(), # Change ggplot2 theme

palette = c("#E7B800","#00AFBB"),

fun = "event",

legend = "none",

legend.title = "Covid-19 diagnose",

legend.labs = c("No", "Yes"),

xlim = c(0, 30), ylim = c(0, 0.55)) + ggtitle("D Cohort adjusted by days in ICU") + xlab("") + ylab("")

splots_3[[4]] <- ggpar(splots_3[[4]],

font.main = c(12, "bold"),

font.legend = c(8, "bold"),

font.tickslab = c(8, "bold"))

fit_23 <- survfit(Surv(Tiempo_ISTU_AC, ISTU_AC) ~ COVID, data = BASE_2)

print(fit_20)

splots_3[[5]] <- ggsurvplot(fit_23,

pval = TRUE,

pval.coord = c(0, 0.35),

pval.size = c(3),

conf.int = TRUE,

risk.table.col = "strata", # Change risk table color by groups

ggtheme = theme_classic(), # Change ggplot2 theme

palette = c("#E7B800","#00AFBB"),

fun = "event",

legend = c(0.7, 0.8),

legend.title = "Covid-19 diagnose",

legend.labs = c("No", "Yes"),

xlim = c(0, 30), ylim = c(0, 0.55)) + ggtitle("E") + xlab("") + ylab("")

splots_3[[5]] <- ggpar(splots_3[[5]],

font.main = c(12, "bold"),

font.legend = c(8, "bold"),

font.tickslab = c(8, "bold"))

fit_24 <- survfit(Surv(Tiempo_NEU, NEU) ~ COVID, data = BASE_2)

print(fit_24)

splots_3[[6]] <- ggsurvplot(fit_24,

pval = TRUE,

pval.coord = c(0, 0.35),

pval.size = c(3),

conf.int = TRUE,

risk.table.col = "strata", # Change risk table color by groups

ggtheme = theme_classic(), # Change ggplot2 theme

palette = c("#E7B800","#00AFBB"),

fun = "event",

legend = "none",

legend.title = "Covid-19 diagnose",

legend.labs = c("No", "Yes"),

xlim = c(0, 30), ylim = c(0, 0.55)) + ggtitle("F") + xlab("Days") + ylab("")

splots_3[[6]] <- ggpar(splots_3[[6]],

font.main = c(12, "bold"),

font.legend = c(8, "bold"),

font.tickslab = c(8, "bold"))

fit_25 <- survfit(Surv(Tiempo_ITS_AC, ITS_AC) ~ COVID, data = BASE_3)

print(fit_25)

splots_3[[7]] <- ggsurvplot(fit_25,

pval = TRUE,

pval.coord = c(0, 0.35),

pval.size = c(3),

conf.int = TRUE,

risk.table.col = "strata", # Change risk table color by groups

ggtheme = theme_classic(), # Change ggplot2 theme

palette = c("#E7B800","#00AFBB"),

fun = "event",

legend = "none",

legend.title = "Covid-19 diagnose",

legend.labs = c("No", "Yes"),

xlim = c(0, 30), ylim = c(0, 0.55)) + ggtitle("G Cohort adjusted by days in ventilation") + xlab("") + ylab("")

splots_3[[7]] <- ggpar(splots_3[[7]],

font.main = c(12, "bold"),

font.legend = c(8, "bold"),

font.tickslab = c(8, "bold"))

fit_26 <- survfit(Surv(Tiempo_ISTU_AC, ISTU_AC) ~ COVID, data = BASE_3)

print(fit_26)

splots_3[[8]] <- ggsurvplot(fit_26,

pval = TRUE,

pval.coord = c(0, 0.35),

pval.size = c(3),

conf.int = TRUE,

risk.table.col = "strata", # Change risk table color by groups

ggtheme = theme_classic(), # Change ggplot2 theme

palette = c("#E7B800","#00AFBB"),

fun = "event",

legend = "none",

legend.title = "Covid-19 diagnose",

legend.labs = c("No", "Yes"),

xlim = c(0, 30), ylim = c(0, 0.55)) + ggtitle("H") + xlab("") + ylab("")

splots_3[[8]] <- ggpar(splots_3[[8]],

font.main = c(12, "bold"),

font.legend = c(8, "bold"),

font.tickslab = c(8, "bold"))

fit_27 <- survfit(Surv(Tiempo_NEU, NEU) ~ COVID, data = BASE_3)

print(fit_27)

splots_3[[9]] <- ggsurvplot(fit_27,

pval = TRUE,

pval.coord = c(0, 0.35),

pval.size = c(3),

conf.int = TRUE,

risk.table.col = "strata", # Change risk table color by groups

ggtheme = theme_classic(), # Change ggplot2 theme

palette = c("#E7B800","#00AFBB"),

fun = "event",

legend = "none",

legend.title = "Covid-19 diagnose",

legend.labs = c("No", "Yes"),

xlim = c(0, 30), ylim = c(0, 0.55)) + ggtitle("I") + xlab("") + ylab("")

splots_3[[9]] <- ggpar(splots_3[[9]],

font.main = c(12, "bold"),

font.legend = c(8, "bold"),

font.tickslab = c(8, "bold"))

plot_3 <- arrange_ggsurvplots(splots_3, print = TRUE, ncol = 3, nrow =3)

#regresión de cox univariable y evaluación de supuestos

cox1 <- coxph(Surv(Tiempo_IAAS, IAAS) ~ COVID, data = BASE)

summary(cox1)

ggcoxdiagnostics(cox1, type = "dfbeta",

linear.predictions = FALSE, ggtheme = theme_bw())

ggcoxdiagnostics(cox1, type = "deviance",

linear.predictions = FALSE, ggtheme = theme_bw())

ph_1 <- cox.zph(cox1)

ggcoxzph(ph_1)

ggcoxzph(ph_1 [1])

cox2 <- coxph(Surv(Tiempo_IAAS, IAAS) ~ EDAD, data = BASE)

summary(cox2)

ggcoxdiagnostics(cox2, type = "dfbeta",

linear.predictions = FALSE, ggtheme = theme_bw())

ggcoxdiagnostics(cox2, type = "deviance",

linear.predictions = FALSE, ggtheme = theme_bw())

ph_2 <- cox.zph(cox2)

ggcoxzph(ph_2)

ggcoxzph(ph_2 [1])

cox3 <- coxph(Surv(Tiempo_IAAS, IAAS) ~ SEXO, data = BASE)

summary(cox3)

ggcoxdiagnostics(cox3, type = "dfbeta",

linear.predictions = FALSE, ggtheme = theme_bw())

ggcoxdiagnostics(cox3, type = "deviance",

linear.predictions = FALSE, ggtheme = theme_bw())

ph_3 <- cox.zph(cox3)

ggcoxzph(ph_3)

ggcoxzph(ph_3 [1])

cox4 <- coxph(Surv(Tiempo_IAAS, IAAS) ~ DÍAS_UCI, data = BASE)

summary(cox4)

ggcoxdiagnostics(cox4, type = "dfbeta",

linear.predictions = FALSE, ggtheme = theme_bw())

ggcoxdiagnostics(cox4, type = "deviance",

linear.predictions = FALSE, ggtheme = theme_bw())

ph_4 <- cox.zph(cox4)

ggcoxzph(ph_4)

ggcoxzph(ph_4 [1])

cox5 <- coxph(Surv(Tiempo_IAAS, IAAS) ~ Tiempo_VENTILACION, data = BASE)

summary(cox5)

ggcoxdiagnostics(cox5, type = "dfbeta",

linear.predictions = FALSE, ggtheme = theme_bw())

ggcoxdiagnostics(cox5, type = "deviance",

linear.predictions = FALSE, ggtheme = theme_bw())

ph_5 <- cox.zph(cox5)

ggcoxzph(ph_5)

ggcoxzph(ph_5 [1])

cox6 <- coxph(Surv(Tiempo_IAAS, IAAS) ~ Neumopatía, data = BASE)

summary(cox6)

ggcoxdiagnostics(cox6, type = "dfbeta",

linear.predictions = FALSE, ggtheme = theme_bw())

ggcoxdiagnostics(cox6, type = "deviance",

linear.predictions = FALSE, ggtheme = theme_bw())

ph_6 <- cox.zph(cox6)

ggcoxzph(ph_6)

ggcoxzph(ph_6 [1])

cox7 <- coxph(Surv(Tiempo_IAAS, IAAS) ~ Diabetes, data = BASE)

summary(cox7)

ggcoxdiagnostics(cox7, type = "dfbeta",

linear.predictions = FALSE, ggtheme = theme_bw())

ggcoxdiagnostics(cox7, type = "deviance",

linear.predictions = FALSE, ggtheme = theme_bw())

ph_7 <- cox.zph(cox7)

ggcoxzph(ph_7)

ggcoxzph(ph_7 [1])

cox8 <- coxph(Surv(Tiempo_IAAS, IAAS) ~ Tabaquismo, data = BASE)

summary(cox8)

ggcoxdiagnostics(cox8, type = "dfbeta",

linear.predictions = FALSE, ggtheme = theme_bw())

ggcoxdiagnostics(cox8, type = "deviance",

linear.predictions = FALSE, ggtheme = theme_bw())

ph_8 <- cox.zph(cox8)

ggcoxzph(ph_8)

ggcoxzph(ph_8 [1])

cox1 <- coxph(Surv(Tiempo_IAAS, IAAS) ~ COVID, data = BASE_2)

summary(cox1)

ggcoxdiagnostics(cox1, type = "dfbeta",

linear.predictions = FALSE, ggtheme = theme_bw())

ggcoxdiagnostics(cox1, type = "deviance",

linear.predictions = FALSE, ggtheme = theme_bw())

ph_1 <- cox.zph(cox1)

ggcoxzph(ph_1)

ggcoxzph(ph_1 [1])

cox2 <- coxph(Surv(Tiempo_IAAS, IAAS) ~ EDAD, data = BASE_2)

summary(cox2)

ggcoxdiagnostics(cox2, type = "dfbeta",

linear.predictions = FALSE, ggtheme = theme_bw())

ggcoxdiagnostics(cox2, type = "deviance",

linear.predictions = FALSE, ggtheme = theme_bw())

ph_2 <- cox.zph(cox2)

ggcoxzph(ph_2)

ggcoxzph(ph_2 [1])

cox3 <- coxph(Surv(Tiempo_IAAS, IAAS) ~ SEXO, data = BASE_2)

summary(cox3)

ggcoxdiagnostics(cox3, type = "dfbeta",

linear.predictions = FALSE, ggtheme = theme_bw())

ggcoxdiagnostics(cox3, type = "deviance",

linear.predictions = FALSE, ggtheme = theme_bw())

ph_3 <- cox.zph(cox3)

ggcoxzph(ph_3)

ggcoxzph(ph_3 [1])

cox4 <- coxph(Surv(Tiempo_IAAS, IAAS) ~ DÍAS_UCI, data = BASE_2)

summary(cox4)

ggcoxdiagnostics(cox4, type = "dfbeta",

linear.predictions = FALSE, ggtheme = theme_bw())

ggcoxdiagnostics(cox4, type = "deviance",

linear.predictions = FALSE, ggtheme = theme_bw())

ph_4 <- cox.zph(cox4)

ggcoxzph(ph_4)

ggcoxzph(ph_4 [1])

cox5 <- coxph(Surv(Tiempo_IAAS, IAAS) ~ Tiempo_VENTILACION, data = BASE_2)

summary(cox5)

ggcoxdiagnostics(cox5, type = "dfbeta",

linear.predictions = FALSE, ggtheme = theme_bw())

ggcoxdiagnostics(cox5, type = "deviance",

linear.predictions = FALSE, ggtheme = theme_bw())

ph_5 <- cox.zph(cox5)

ggcoxzph(ph_5)

ggcoxzph(ph_5 [1])

cox6 <- coxph(Surv(Tiempo_IAAS, IAAS) ~ Neumopatía, data = BASE_2)

summary(cox6)

ggcoxdiagnostics(cox6, type = "dfbeta",

linear.predictions = FALSE, ggtheme = theme_bw())

ggcoxdiagnostics(cox6, type = "deviance",

linear.predictions = FALSE, ggtheme = theme_bw())

ph_6 <- cox.zph(cox6)

ggcoxzph(ph_6)

ggcoxzph(ph_6 [1])

cox7 <- coxph(Surv(Tiempo_IAAS, IAAS) ~ Diabetes, data = BASE_2)

summary(cox7)

ggcoxdiagnostics(cox7, type = "dfbeta",

linear.predictions = FALSE, ggtheme = theme_bw())

ggcoxdiagnostics(cox7, type = "deviance",

linear.predictions = FALSE, ggtheme = theme_bw())

ph_7 <- cox.zph(cox7)

ggcoxzph(ph_7)

ggcoxzph(ph_7 [1])

cox8 <- coxph(Surv(Tiempo_IAAS, IAAS) ~ Tabaquismo, data = BASE_2)

summary(cox8)

ggcoxdiagnostics(cox8, type = "dfbeta",

linear.predictions = FALSE, ggtheme = theme_bw())

ggcoxdiagnostics(cox8, type = "deviance",

linear.predictions = FALSE, ggtheme = theme_bw())

ph_8 <- cox.zph(cox8)

ggcoxzph(ph_8)

ggcoxzph(ph_8 [1])

cox1 <- coxph(Surv(Tiempo_IAAS, IAAS) ~ COVID, data = BASE_3)

summary(cox1)

ggcoxdiagnostics(cox1, type = "dfbeta",

linear.predictions = FALSE, ggtheme = theme_bw())

ggcoxdiagnostics(cox1, type = "deviance",

linear.predictions = FALSE, ggtheme = theme_bw())

ph_1 <- cox.zph(cox1)

ggcoxzph(ph_1)

ggcoxzph(ph_1 [1])

cox2 <- coxph(Surv(Tiempo_IAAS, IAAS) ~ EDAD, data = BASE_3)

summary(cox2)

ggcoxdiagnostics(cox2, type = "dfbeta",

linear.predictions = FALSE, ggtheme = theme_bw())

ggcoxdiagnostics(cox2, type = "deviance",

linear.predictions = FALSE, ggtheme = theme_bw())

ph_2 <- cox.zph(cox2)

ggcoxzph(ph_2)

ggcoxzph(ph_2 [1])

cox3 <- coxph(Surv(Tiempo_IAAS, IAAS) ~ SEXO, data = BASE_3)

summary(cox3)

ggcoxdiagnostics(cox3, type = "dfbeta",

linear.predictions = FALSE, ggtheme = theme_bw())

ggcoxdiagnostics(cox3, type = "deviance",

linear.predictions = FALSE, ggtheme = theme_bw())

ph_3 <- cox.zph(cox3)

ggcoxzph(ph_3)

ggcoxzph(ph_3 [1])

cox4 <- coxph(Surv(Tiempo_IAAS, IAAS) ~ DÍAS_UCI, data = BASE_3)

summary(cox4)

ggcoxdiagnostics(cox4, type = "dfbeta",

linear.predictions = FALSE, ggtheme = theme_bw())

ggcoxdiagnostics(cox4, type = "deviance",

linear.predictions = FALSE, ggtheme = theme_bw())

ph_4 <- cox.zph(cox4)

ggcoxzph(ph_4)

ggcoxzph(ph_4 [1])

cox5 <- coxph(Surv(Tiempo_IAAS, IAAS) ~ Tiempo_VENTILACION, data = BASE_3)

summary(cox5)

ggcoxdiagnostics(cox5, type = "dfbeta",

linear.predictions = FALSE, ggtheme = theme_bw())

ggcoxdiagnostics(cox5, type = "deviance",

linear.predictions = FALSE, ggtheme = theme_bw())

ph_5 <- cox.zph(cox5)

ggcoxzph(ph_5)

ggcoxzph(ph_5 [1])

cox6 <- coxph(Surv(Tiempo_IAAS, IAAS) ~ Neumopatía, data = BASE_3)

summary(cox6)

ggcoxdiagnostics(cox6, type = "dfbeta",

linear.predictions = FALSE, ggtheme = theme_bw())

ggcoxdiagnostics(cox6, type = "deviance",

linear.predictions = FALSE, ggtheme = theme_bw())

ph_6 <- cox.zph(cox6)

ggcoxzph(ph_6)

ggcoxzph(ph_6 [1])

cox7 <- coxph(Surv(Tiempo_IAAS, IAAS) ~ Diabetes, data = BASE_3)

summary(cox7)

ggcoxdiagnostics(cox7, type = "dfbeta",

linear.predictions = FALSE, ggtheme = theme_bw())

ggcoxdiagnostics(cox7, type = "deviance",

linear.predictions = FALSE, ggtheme = theme_bw())

ph_7 <- cox.zph(cox7)

ggcoxzph(ph_7)

ggcoxzph(ph_7 [1])

cox8 <- coxph(Surv(Tiempo_IAAS, IAAS) ~ Tabaquismo, data = BASE_3)

summary(cox8)

ggcoxdiagnostics(cox8, type = "dfbeta",

linear.predictions = FALSE, ggtheme = theme_bw())

ggcoxdiagnostics(cox8, type = "deviance",

linear.predictions = FALSE, ggtheme = theme_bw())

ph_8 <- cox.zph(cox8)

ggcoxzph(ph_8)

ggcoxzph(ph_8 [1])

cox1 <- coxph(Surv(Tiempo_NEU, NEU) ~ COVID, data = BASE)

summary(cox1)

ggcoxdiagnostics(cox1, type = "dfbeta",

linear.predictions = FALSE, ggtheme = theme_bw())

ggcoxdiagnostics(cox1, type = "deviance",

linear.predictions = FALSE, ggtheme = theme_bw())

ph_1 <- cox.zph(cox1)

ggcoxzph(ph_1)

ggcoxzph(ph_1 [1])

cox2 <- coxph(Surv(Tiempo_NEU, NEU) ~ EDAD, data = BASE)

summary(cox2)

ggcoxdiagnostics(cox2, type = "dfbeta",

linear.predictions = FALSE, ggtheme = theme_bw())

ggcoxdiagnostics(cox2, type = "deviance",

linear.predictions = FALSE, ggtheme = theme_bw())

ph_2 <- cox.zph(cox2)

ggcoxzph(ph_2)

ggcoxzph(ph_2 [1])

cox3 <- coxph(Surv(Tiempo_NEU, NEU) ~ SEXO, data = BASE)

summary(cox3)

ggcoxdiagnostics(cox3, type = "dfbeta",

linear.predictions = FALSE, ggtheme = theme_bw())

ggcoxdiagnostics(cox3, type = "deviance",

linear.predictions = FALSE, ggtheme = theme_bw())

ph_3 <- cox.zph(cox3)

ggcoxzph(ph_3)

ggcoxzph(ph_3 [1])

cox4 <- coxph(Surv(Tiempo_NEU, NEU) ~ DÍAS_UCI, data = BASE)

summary(cox4)

ggcoxdiagnostics(cox4, type = "dfbeta",

linear.predictions = FALSE, ggtheme = theme_bw())

ggcoxdiagnostics(cox4, type = "deviance",

linear.predictions = FALSE, ggtheme = theme_bw())

ph_4 <- cox.zph(cox4)

ggcoxzph(ph_4)

ggcoxzph(ph_4 [1])

cox5 <- coxph(Surv(Tiempo_NEU, NEU) ~ Tiempo_VENTILACION, data = BASE)

summary(cox5)

ggcoxdiagnostics(cox5, type = "dfbeta",

linear.predictions = FALSE, ggtheme = theme_bw())

ggcoxdiagnostics(cox5, type = "deviance",

linear.predictions = FALSE, ggtheme = theme_bw())

ph_5 <- cox.zph(cox5)

ggcoxzph(ph_5)

ggcoxzph(ph_5 [1])

cox6 <- coxph(Surv(Tiempo_NEU, NEU) ~ Neumopatía, data = BASE)

summary(cox6)

ggcoxdiagnostics(cox6, type = "dfbeta",

linear.predictions = FALSE, ggtheme = theme_bw())

ggcoxdiagnostics(cox6, type = "deviance",

linear.predictions = FALSE, ggtheme = theme_bw())

ph_6 <- cox.zph(cox6)

ggcoxzph(ph_6)

ggcoxzph(ph_6 [1])

cox7 <- coxph(Surv(Tiempo_NEU, NEU) ~ Diabetes, data = BASE)

summary(cox7)

ggcoxdiagnostics(cox7, type = "dfbeta",

linear.predictions = FALSE, ggtheme = theme_bw())

ggcoxdiagnostics(cox7, type = "deviance",

linear.predictions = FALSE, ggtheme = theme_bw())

ph_7 <- cox.zph(cox7)

ggcoxzph(ph_7)

ggcoxzph(ph_7 [1])

cox8 <- coxph(Surv(Tiempo_NEU, NEU) ~ Tabaquismo, data = BASE)

summary(cox8)

ggcoxdiagnostics(cox8, type = "dfbeta",

linear.predictions = FALSE, ggtheme = theme_bw())

ggcoxdiagnostics(cox8, type = "deviance",

linear.predictions = FALSE, ggtheme = theme_bw())

ph_8 <- cox.zph(cox8)

ggcoxzph(ph_8)

ggcoxzph(ph_8 [1])

cox1 <- coxph(Surv(Tiempo_NEU, NEU) ~ COVID, data = BASE_2)

summary(cox1)

ggcoxdiagnostics(cox1, type = "dfbeta",

linear.predictions = FALSE, ggtheme = theme_bw())

ggcoxdiagnostics(cox1, type = "deviance",

linear.predictions = FALSE, ggtheme = theme_bw())

ph_1 <- cox.zph(cox1)

ggcoxzph(ph_1)

ggcoxzph(ph_1 [1])

cox2 <- coxph(Surv(Tiempo_NEU, NEU) ~ EDAD, data = BASE_2)

summary(cox2)

ggcoxdiagnostics(cox2, type = "dfbeta",

linear.predictions = FALSE, ggtheme = theme_bw())

ggcoxdiagnostics(cox2, type = "deviance",

linear.predictions = FALSE, ggtheme = theme_bw())

ph_2 <- cox.zph(cox2)

ggcoxzph(ph_2)

ggcoxzph(ph_2 [1])

cox3 <- coxph(Surv(Tiempo_NEU, NEU) ~ SEXO, data = BASE_2)

summary(cox3)

ggcoxdiagnostics(cox3, type = "dfbeta",

linear.predictions = FALSE, ggtheme = theme_bw())

ggcoxdiagnostics(cox3, type = "deviance",

linear.predictions = FALSE, ggtheme = theme_bw())

ph_3 <- cox.zph(cox3)

ggcoxzph(ph_3)

ggcoxzph(ph_3 [1])

cox4 <- coxph(Surv(Tiempo_NEU, NEU) ~ DÍAS_UCI, data = BASE_2)

summary(cox4)

ggcoxdiagnostics(cox4, type = "dfbeta",

linear.predictions = FALSE, ggtheme = theme_bw())

ggcoxdiagnostics(cox4, type = "deviance",

linear.predictions = FALSE, ggtheme = theme_bw())

ph_4 <- cox.zph(cox4)

ggcoxzph(ph_4)

ggcoxzph(ph_4 [1])

cox5 <- coxph(Surv(Tiempo_NEU, NEU) ~ Tiempo_VENTILACION, data = BASE_2)

summary(cox5)

ggcoxdiagnostics(cox5, type = "dfbeta",

linear.predictions = FALSE, ggtheme = theme_bw())

ggcoxdiagnostics(cox5, type = "deviance",

linear.predictions = FALSE, ggtheme = theme_bw())

ph_5 <- cox.zph(cox5)

ggcoxzph(ph_5)

ggcoxzph(ph_5 [1])

cox6 <- coxph(Surv(Tiempo_NEU, NEU) ~ Neumopatía, data = BASE_2)

summary(cox6)

ggcoxdiagnostics(cox6, type = "dfbeta",

linear.predictions = FALSE, ggtheme = theme_bw())

ggcoxdiagnostics(cox6, type = "deviance",

linear.predictions = FALSE, ggtheme = theme_bw())

ph_6 <- cox.zph(cox6)

ggcoxzph(ph_6)

ggcoxzph(ph_6 [1])

cox7 <- coxph(Surv(Tiempo_NEU, NEU) ~ Diabetes, data = BASE_2)

summary(cox7)

ggcoxdiagnostics(cox7, type = "dfbeta",

linear.predictions = FALSE, ggtheme = theme_bw())

ggcoxdiagnostics(cox7, type = "deviance",

linear.predictions = FALSE, ggtheme = theme_bw())

ph_7 <- cox.zph(cox7)

ggcoxzph(ph_7)

ggcoxzph(ph_7 [1])

cox8 <- coxph(Surv(Tiempo_NEU, NEU) ~ Tabaquismo, data = BASE_2)

summary(cox8)

ggcoxdiagnostics(cox8, type = "dfbeta",

linear.predictions = FALSE, ggtheme = theme_bw())

ggcoxdiagnostics(cox8, type = "deviance",

linear.predictions = FALSE, ggtheme = theme_bw())

ph_8 <- cox.zph(cox8)

ggcoxzph(ph_8)

ggcoxzph(ph_8 [1])

cox1 <- coxph(Surv(Tiempo_NEU, NEU) ~ COVID, data = BASE_3)

summary(cox1)

ggcoxdiagnostics(cox1, type = "dfbeta",

linear.predictions = FALSE, ggtheme = theme_bw())

ggcoxdiagnostics(cox1, type = "deviance",

linear.predictions = FALSE, ggtheme = theme_bw())

ph_1 <- cox.zph(cox1)

ggcoxzph(ph_1)

ggcoxzph(ph_1 [1])

cox2 <- coxph(Surv(Tiempo_NEU, NEU) ~ EDAD, data = BASE_3)

summary(cox2)

ggcoxdiagnostics(cox2, type = "dfbeta",

linear.predictions = FALSE, ggtheme = theme_bw())

ggcoxdiagnostics(cox2, type = "deviance",

linear.predictions = FALSE, ggtheme = theme_bw())

ph_2 <- cox.zph(cox2)

ggcoxzph(ph_2)

ggcoxzph(ph_2 [1])

cox3 <- coxph(Surv(Tiempo_NEU, NEU) ~ SEXO, data = BASE_3)

summary(cox3)

ggcoxdiagnostics(cox3, type = "dfbeta",

linear.predictions = FALSE, ggtheme = theme_bw())

ggcoxdiagnostics(cox3, type = "deviance",

linear.predictions = FALSE, ggtheme = theme_bw())

ph_3 <- cox.zph(cox3)

ggcoxzph(ph_3)

ggcoxzph(ph_3 [1])

cox4 <- coxph(Surv(Tiempo_NEU, NEU) ~ DÍAS_UCI, data = BASE_3)

summary(cox4)

ggcoxdiagnostics(cox4, type = "dfbeta",

linear.predictions = FALSE, ggtheme = theme_bw())

ggcoxdiagnostics(cox4, type = "deviance",

linear.predictions = FALSE, ggtheme = theme_bw())

ph_4 <- cox.zph(cox4)

ggcoxzph(ph_4)

ggcoxzph(ph_4 [1])

cox5 <- coxph(Surv(Tiempo_NEU, NEU) ~ Tiempo_VENTILACION, data = BASE_3)

summary(cox5)

ggcoxdiagnostics(cox5, type = "dfbeta",

linear.predictions = FALSE, ggtheme = theme_bw())

ggcoxdiagnostics(cox5, type = "deviance",

linear.predictions = FALSE, ggtheme = theme_bw())

ph_5 <- cox.zph(cox5)

ggcoxzph(ph_5)

ggcoxzph(ph_5 [1])

cox6 <- coxph(Surv(Tiempo_NEU, NEU) ~ Neumopatía, data = BASE_3)

summary(cox6)

ggcoxdiagnostics(cox6, type = "dfbeta",

linear.predictions = FALSE, ggtheme = theme_bw())

ggcoxdiagnostics(cox6, type = "deviance",

linear.predictions = FALSE, ggtheme = theme_bw())

ph_6 <- cox.zph(cox6)

ggcoxzph(ph_6)

ggcoxzph(ph_6 [1])

cox7 <- coxph(Surv(Tiempo_NEU, NEU) ~ Diabetes, data = BASE_3)

summary(cox7)

ggcoxdiagnostics(cox7, type = "dfbeta",

linear.predictions = FALSE, ggtheme = theme_bw())

ggcoxdiagnostics(cox7, type = "deviance",

linear.predictions = FALSE, ggtheme = theme_bw())

ph_7 <- cox.zph(cox7)

ggcoxzph(ph_7)

ggcoxzph(ph_7 [1])

cox8 <- coxph(Surv(Tiempo_NEU, NEU) ~ Tabaquismo, data = BASE_3)

summary(cox8)

ggcoxdiagnostics(cox8, type = "dfbeta",

linear.predictions = FALSE, ggtheme = theme_bw())

ggcoxdiagnostics(cox8, type = "deviance",

linear.predictions = FALSE, ggtheme = theme_bw())

ph_8 <- cox.zph(cox8)

ggcoxzph(ph_8)

ggcoxzph(ph_8 [1])

#regresión de cox multivariable y evaluación de supuestos

res.cox_1 <- coxph(Surv(Tiempo_IAAS, IAAS) ~ COVID + EDAD + SEXO + DÍAS_UCI + Tiempo_VENTILACION + Neumopatía + Diabetes + Tabaquismo, data = BASE)

summary(res.cox_1)

extractAIC(res.cox_1)

ggcoxdiagnostics(res.cox_1, type = "dfbeta",

linear.predictions = FALSE, ggtheme = theme_bw())

ggcoxdiagnostics(res.cox_1, type = "deviance",

linear.predictions = FALSE, ggtheme = theme_bw())

test.ph_1 <- cox.zph(res.cox_1)

ggcoxzph(test.ph_1)

ggcoxzph(test.ph_1 [1])

ggcoxzph(test.ph_1 [2])

ggcoxzph(test.ph_1 [3])

ggcoxzph(test.ph_1 [4])

ggcoxzph(test.ph_1 [5])

ggcoxzph(test.ph_1 [6])

ggcoxzph(test.ph_1 [7])

ggcoxzph(test.ph_1 [8])

res.cox_1 <- coxph(Surv(Tiempo_IAAS, IAAS) ~ COVID + EDAD + SEXO + DÍAS_UCI + Tiempo_VENTILACION + Neumopatía + Diabetes + Tabaquismo + frailty (ID,

distribution = "gaussian", sparse = FALSE, method = "reml"), data = BASE)

summary(res.cox_1)

res.cox_2 <- coxph(Surv(Tiempo_IAAS, IAAS) ~ COVID + EDAD + SEXO + DÍAS_UCI + Tiempo_VENTILACION + Neumopatía + Diabetes + Tabaquismo, data = BASE_2)

summary(res.cox_2)

extractAIC(res.cox_2)

ggcoxdiagnostics(res.cox_2, type = "dfbeta",

linear.predictions = FALSE, ggtheme = theme_bw())

ggcoxdiagnostics(res.cox_2, type = "deviance",

linear.predictions = FALSE, ggtheme = theme_bw())

test.ph_2 <- cox.zph(res.cox_2)

ggcoxzph(test.ph_2)

ggcoxzph(test.ph_2 [1])

ggcoxzph(test.ph_2 [2])

ggcoxzph(test.ph_2 [3])

ggcoxzph(test.ph_2 [4])

ggcoxzph(test.ph_2 [5])

ggcoxzph(test.ph_2 [6])

ggcoxzph(test.ph_2 [7])

ggcoxzph(test.ph_2 [8])

res.cox_2 <- coxph(Surv(Tiempo_IAAS, IAAS) ~ COVID + EDAD + SEXO + DÍAS_UCI + Tiempo_VENTILACION + Neumopatía + Diabetes + Tabaquismo + frailty (ID,

distribution = "gaussian", sparse = FALSE, method = "reml"), data = BASE_2)

summary(res.cox_2)

res.cox_3 <- coxph(Surv(Tiempo_IAAS, IAAS) ~ COVID + EDAD + SEXO + DÍAS_UCI + Tiempo_VENTILACION + Neumopatía + Diabetes + Tabaquismo, data = BASE_3)

summary(res.cox_3)

extractAIC(res.cox_3)

ggcoxdiagnostics(res.cox_3, type = "dfbeta",

linear.predictions = FALSE, ggtheme = theme_bw())

ggcoxdiagnostics(res.cox_3, type = "deviance",

linear.predictions = FALSE, ggtheme = theme_bw())

test.ph_3 <- cox.zph(res.cox_3)

ggcoxzph(test.ph_3)

ggcoxzph(test.ph_3 [1])

ggcoxzph(test.ph_3 [2])

ggcoxzph(test.ph_3 [3])

ggcoxzph(test.ph_3 [4])

ggcoxzph(test.ph_3 [5])

ggcoxzph(test.ph_3 [6])

ggcoxzph(test.ph_3 [7])

ggcoxzph(test.ph_3 [8])

res.cox_3 <- coxph(Surv(Tiempo_IAAS, IAAS) ~ COVID + EDAD + SEXO + DÍAS_UCI + Tiempo_VENTILACION + Neumopatía + Diabetes + Tabaquismo + frailty (ID,

distribution = "gaussian", sparse = FALSE, method = "reml"), data = BASE_3)

summary(res.cox_3)

res.cox_4 <- coxph(Surv(Tiempo_NEU, NEU) ~ COVID + EDAD + SEXO + DÍAS_UCI + Tiempo_VENTILACION + Neumopatía + Diabetes + Tabaquismo, data = BASE)

summary(res.cox_4)

extractAIC(res.cox_4)

ggcoxdiagnostics(res.cox_4, type = "dfbeta",

linear.predictions = FALSE, ggtheme = theme_bw())

ggcoxdiagnostics(res.cox_4, type = "deviance",

linear.predictions = FALSE, ggtheme = theme_bw())

test.ph_4 <- cox.zph(res.cox_4)

ggcoxzph(test.ph_4)

ggcoxzph(test.ph_4 [1])

ggcoxzph(test.ph_4 [2])

ggcoxzph(test.ph_4 [3])

ggcoxzph(test.ph_4 [4])

ggcoxzph(test.ph_4 [5])

ggcoxzph(test.ph_4 [6])

ggcoxzph(test.ph_4 [7])

ggcoxzph(test.ph_4 [8])

res.cox_5 <- coxph(Surv(Tiempo_NEU, NEU) ~ COVID + EDAD + SEXO + DÍAS_UCI + Tiempo_VENTILACION + Neumopatía + Diabetes + Tabaquismo, data = BASE_2)

summary(res.cox_5)

extractAIC(res.cox_5)

ggcoxdiagnostics(res.cox_5, type = "dfbeta",

linear.predictions = FALSE, ggtheme = theme_bw())

ggcoxdiagnostics(res.cox_5, type = "deviance",

linear.predictions = FALSE, ggtheme = theme_bw())

test.ph_5 <- cox.zph(res.cox_5)

ggcoxzph(test.ph_5)

ggcoxzph(test.ph_5 [1])

ggcoxzph(test.ph_5 [2])

ggcoxzph(test.ph_5 [3])

ggcoxzph(test.ph_5 [4])

ggcoxzph(test.ph_5 [5])

ggcoxzph(test.ph_5 [6])

ggcoxzph(test.ph_5 [7])

ggcoxzph(test.ph_5 [8])

res.cox_6 <- coxph(Surv(Tiempo_NEU, NEU) ~ COVID + EDAD + SEXO + DÍAS_UCI + Tiempo_VENTILACION + Neumopatía + Diabetes + Tabaquismo, data = BASE_3)

summary(res.cox_6)

extractAIC(res.cox_6)

ggcoxdiagnostics(res.cox_6, type = "dfbeta",

linear.predictions = FALSE, ggtheme = theme_bw())

ggcoxdiagnostics(res.cox_6, type = "deviance",

linear.predictions = FALSE, ggtheme = theme_bw())

test.ph_6 <- cox.zph(res.cox_6)

ggcoxzph(test.ph_6)

ggcoxzph(test.ph_6 [1])

ggcoxzph(test.ph_6 [2])

ggcoxzph(test.ph_6 [3])

ggcoxzph(test.ph_6 [4])

ggcoxzph(test.ph_6 [5])

ggcoxzph(test.ph_6 [6])

ggcoxzph(test.ph_6 [7])

ggcoxzph(test.ph_6 [8])

ggcoxfunctional(Surv(Tiempo_IAAS, IAAS) ~ EDAD + log(EDAD) + sqrt(EDAD), data = BASE)

ggcoxfunctional(Surv(Tiempo_IAAS, IAAS) ~ DÍAS_UCI + log(DÍAS_UCI) + sqrt(DÍAS_UCI), data = BASE)

ggcoxfunctional(Surv(Tiempo_IAAS, IAAS) ~ Tiempo_VENTILACION + log(Tiempo_VENTILACION) + sqrt(Tiempo_VENTILACION), data = BASE)
